# Porous coordination polymers with ubiquitous and biocompatible metals and a neutral bridging ligand

**DOI:** 10.1038/ncomms6851

**Published:** 2015-01-16

**Authors:** Shin-ichiro Noro, Junya Mizutani, Yuh Hijikata, Ryotaro Matsuda, Hiroshi Sato, Susumu Kitagawa, Kunihisa Sugimoto, Yasutaka Inubushi, Kazuya Kubo, Takayoshi Nakamura

**Affiliations:** 1 Research Institute for Electronic Science, Hokkaido University, Sapporo 001-0020, Japan; 2 Graduate School of Environmental Earth Science, Hokkaido University, Sapporo 060-0810, Japan; 3 PRESTO, Japan Science and Technology Agency (JST), 4-1-8 Honcho, Kawaguchi 332-0012, Japan; 4 Creative Research Institute (CRIS), Hokkaido University, Sapporo 001-0021, Japan; 5 Institute of Transformative Bio-Molecules (WPI-ITbM), Nagoya University, Chikusa-ku, Nagoya 464-8602, Japan; 6 Institute for Integrated Cell-Material Science (WPI-iCeMS), Kyoto University, Kyoto 615-8510, Japan; 7 Research and Utilization Division, Japan Synchrotron Radiation Research Institute, Hyogo 679-5198, Japan; 8 Synthesis Research Laboratory, Kurashiki Research Center, Kuraray Co. Ltd., Sakazu, Kurashiki, Okayama 710-0801, Japan

## Abstract

The design of inexpensive and less toxic porous coordination polymers (PCPs) that show selective adsorption or high adsorption capacity is a critical issue in research on applicable porous materials. Although use of Group II magnesium(II) and calcium(II) ions as building blocks could provide cheaper materials and lead to enhanced biocompatibility, examples of magnesium(II) and calcium(II) PCPs are extremely limited compared with commonly used transition metal ones, because neutral bridging ligands have not been available for magnesium(II) and calcium(II) ions. Here we report a rationally designed neutral and charge-polarized bridging ligand as a new partner for magnesium(II) and calcium(II) ions. The three-dimensional magnesium(II) and calcium(II) PCPs synthesized using such a neutral ligand are stable and show selective adsorption and separation of carbon dioxide over methane at ambient temperature. This synthetic approach allows the structural diversification of Group II magnesium(II) and calcium(II) PCPs.

## Abstract (web summary)

Inexpensive porous materials synthesized from Group II metals may be useful for industrial applications. Here, the authors demonstrate that neutral bridging ligands can be used for the synthesis of magnesium(II) and calcium(II) porous coordination polymers.

## Graphical abstract

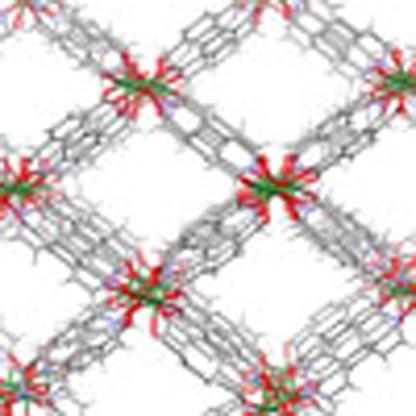

Porous coordination polymers (PCPs) or metal-organic frameworks (MOFs) constructed from metal ions and organic bridging ligands have attracted much attention as intriguing porous materials because of their high structural regularity and diversity, easy modification of frameworks, high porosity and structural flexibility^1^,^2^,^3^,^4^,^5^,^6^,^7^,^8^,^9^,^10^,^11^,^12^,^13^. One of the main trends in work on these materials is the development and improvement of their porous functions, such as storage^14^,^15^, separation^16^,^17^,^18^ and catalysis^19^,^20^,^21^. Towards industrial and biological applications, however, we must consider costs and safety of PCPs in addition to their porous performances. Generally, first-transition-row divalent metal ions such as Co(II), Ni(II), Cu(II) and Zn(II) have been utilized as metal sources of PCPs. On the other hand, Group II metal ions, Mg(II) and Ca(II), belonging to the light metal ions are in abundant supply on the Earth and biofriendly^22^,^23^. If Group II Mg(II) or Ca(II) ions are embedded in PCP frameworks instead of first-transition-row metal ions, the resulting PCPs could become cheaper and have higher biocompatibility.

Nevertheless, the examples of Mg(II) and Ca(II) PCPs are extremely limited to date compared with transition metal PCPs^23^,^24^,^25^,^26^,^27^,^28^,^29^, because neutral bridging ligands have not been available for the construction of Group II Mg(II) and Ca(II) PCPs. Transition metal ions are classed as medium acids according to the hard and soft acids and bases theory. According to this theory, a variety of organic bridging ligands containing anionic carboxylate or imidazolate, and neutral pyridine or imidazole units, tend to be coordinated to them, which makes the PCP frameworks diversified. Furthermore, the combination of anionic and neutral organic bridging ligands is much more effective in diversifying transition metal PCP frameworks. By contrast, Mg(II) and Ca(II) ions are hard acids and, therefore, organic ligands with hard basicity, that is, anionic multicarboxylates have been their sole partners. This is one of the reasons why Mg(II) and Ca(II) PCPs have not been thoroughly investigated.

To overcome this problem, we introduce a new partner, 4,4′-bipyridine-*N*,*N*′-dioxide (bpdo), which is a neutral and charge-polarized bridging ligand with pyridine-*N*-oxide (pyo) moieties, to Group II Mg(II) and Ca(II) ions. Although the structure of the neutral bpdo ligand is similar to that of the neutral ligand 4,4′-bipyridine (4,4′-bpy), we expect that it can coordinate to hard Group II Mg(II) and Ca(II) ions through negatively charged oxygen atoms with hard basicity, despite a totally neutral ligand (Fig. 1a). In this study, using the neutral and charge-polarized bridging ligand bpdo, we demonstrate rational design and syntheses of Mg(II) and Ca(II) PCPs connected by neutral organic ligands. The resulting Mg(II) and Ca(II) PCPs, which are the uncommon examples including neutral bridging ligands, are stable after the removal of guest molecules and show selective adsorption and separation of CO_2_ over CH_4_ at ambient temperature. Our finding contributes to the structural diversification of PCP frameworks with ubiquitous and biocompatible metals.

## Results

### Charge distribution

**Figure 1:**
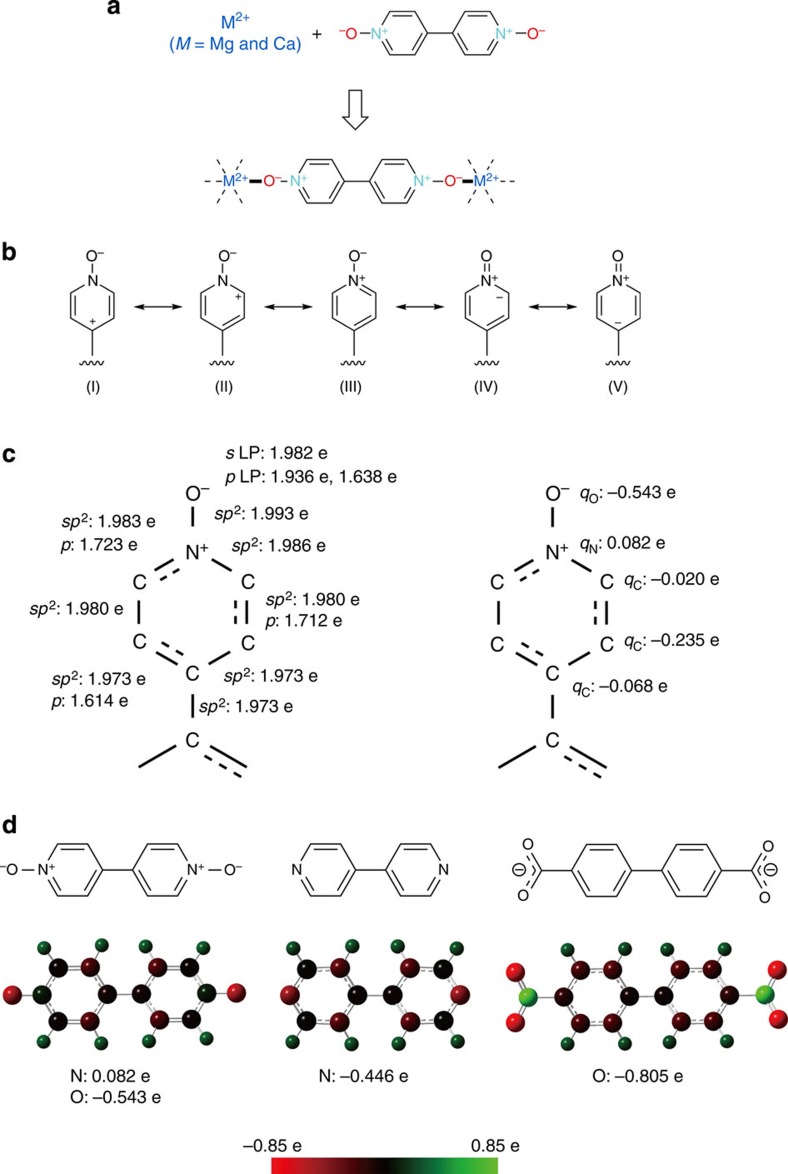
Designing organic ligands for magnesium(II) and calcium(II) porous coordination polymer. (**a**) New neutral partner, 4,4′-bipyridine-*N*,*N*′-dioxide (bpdo), for hard Group II Mg(II) and Ca(II) ions. (**b**) Five resonance structures in a pyridine-*N*-oxide derivative. (**c**) Electron occupation numbers for each bond and natural bond orbital (NBO) charges for each atom in bpdo. (**d**) NBO charges for selected atoms in organic bpdo, 4,4′-bipyridine and 4,4′-biphenyldicarboxylate ligands.

First, we evaluated the hard basicity of the bpdo ligand. Generally, the bond between a hard acid and a hard base is dominated by an ionic interaction. The bpdo ligand has two coordinated oxygen atoms, which are better coordination sites for hard acids than nitrogen atoms because of their larger negative charge. We calculated the charge distribution of a bpdo ligand with other typical and similar organic ligands, pyo, 4,4′-bpy, benzoate (bza^−^) and 4,4′-biphenyldicarboxylate (4,4′-bpdc^2−^) to compare the strengths of ionic interactions. It is well known that pyo and their derivatives show a variety of resonance structures. We investigated which resonance structures mainly contribute to the actual structure. Figure 1b shows five resonance structures of a pyo derivative and Fig. 1c shows electron occupation numbers for each bond and natural bond orbital (NBO) charges for each atom in bpdo, which were obtained by the density functional theory. The negative charges are localized on the oxygen atoms, and little π-bond character is observed between the nitrogen and oxygen atoms, supporting the main contribution of the resonance structures (I), (II) and (III). Figure 1d and Supplementary Fig. 1 show NBO charges. The oxygen atoms of the neutral bpdo and pyo ligands have negative charges of −0.543 e and −0.568 e, respectively, which are smaller than those for anionic carboxylate-type bza^−^ and 4,4′-bpdc^2−^ ligands (−0.795 e and −0.805 e, respectively) but larger than the negative charges of the nitrogen atoms of the neutral 4,4′-bpy ligand (−0.446 e). All calculation results conclude that the neutral ligands with pyo moieties should act as stronger hard bases than 4,4′-bpy and be good partners for hard Group II Mg(II) and Ca(II) ions.

### Structural characterization

**Figure 2:**
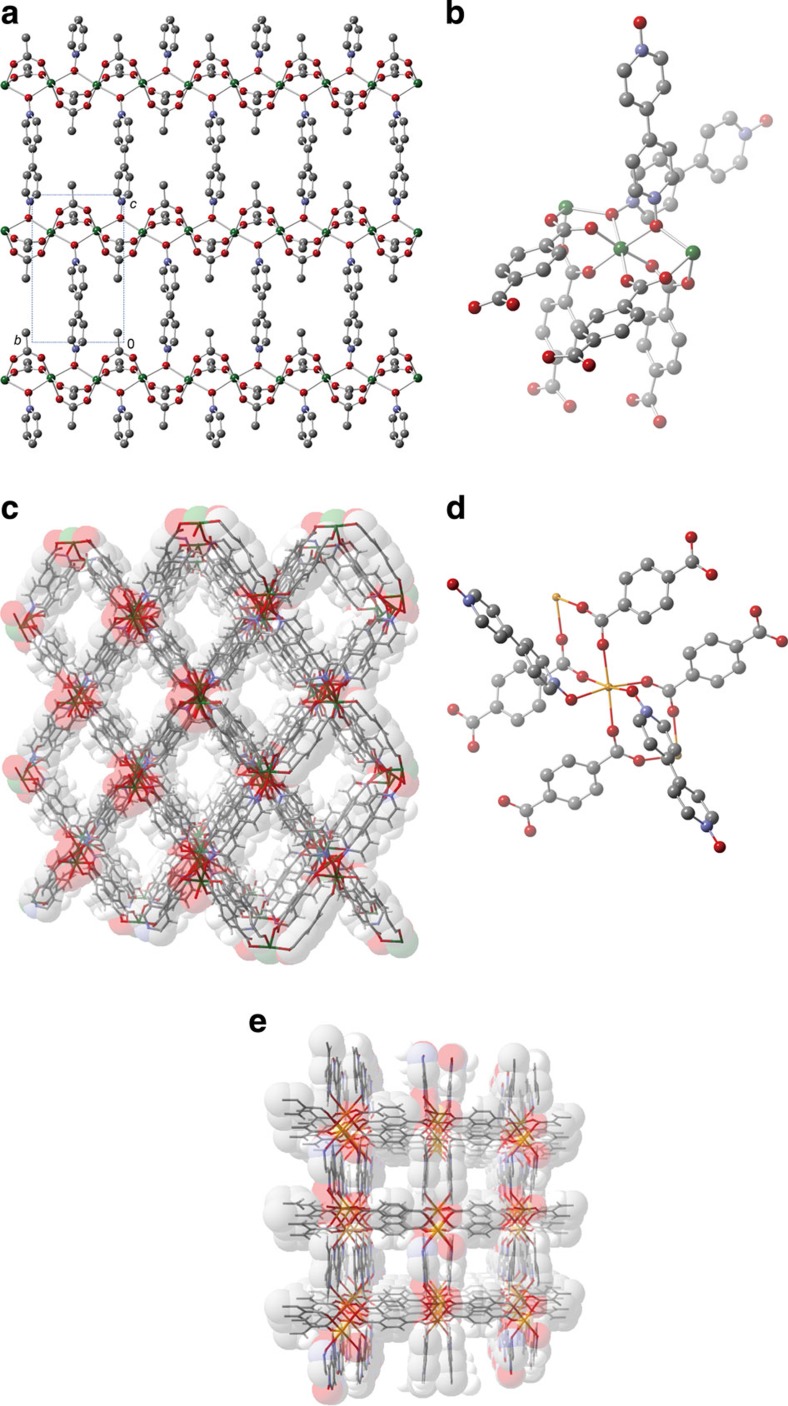
Crystal structures. (**a**) Two-dimensional structure of **1**. (**b**) Coordination environment around the Mg(II) centre in **2**⊃2DMF (DMF=*N*,*N*-dimethylformamide). (**c**) Porous structure in **2**⊃2DMF (the guest DMF molecules are omitted). (**d**) Coordination environment around the Ca(II) centre in **3**⊃0.5DMF. (**e**) Porous structure in **3**⊃0.5DMF (the guest DMF molecules are omitted). In **a**,**b** and **d**, the hydrogen atoms are omitted for clarity. Green represents magnesium; orange, calcium; red, oxygen; blue, nitrogen; grey, carbon and white, hydrogen.

To experimentally prove this new strategy, we next attempted to synthesize Group II Mg(II) and Ca(II) coordination polymers using the neutral bpdo ligand. The reaction of Mg(AcO)_2_·4H_2_O with bpdo yielded the two-dimensional coordination polymer [Mg_2_(AcO)_4_(bpdo)]_*n*_ (**1**). Further, the reaction of MgCl_2_·6H_2_O or Ca(NO_3_)_2_·4H_2_O with 1,4-benzenedicarboxylic acid (1,4-H_2_bdc) and bpdo in *N*,*N*-dimethylformamide (DMF) afforded the three-dimensional PCPs, {[Mg_2_(1,4-bdc)_2_(bpdo)]·2DMF}_*n*_ (**2**⊃2DMF) and {[Ca(1,4-bdc)(bpdo)]·0.5DMF}_*n*_ (**3**⊃0.5DMF). As expected, **1**, **2**⊃2DMF and **3**⊃0.5DMF included the neutral bpdo ligands that bridge the metal centres. The crystal structures of **1**, **2**⊃2DMF and **3**⊃0.5DMF were determined using single-crystal X-ray crystallography at 173 K. **1** has an octahedral Mg(II) environment with four AcO^−^ oxygen atoms and two bpdo oxygen atoms. The Mg–O(AcO^−^) bond distances (2.017(6)–2.085(6) Å) are slightly shorter than the Mg–O(bpdo) bond distances (2.154(4) and 2.161(4) Å). The Mg(II) centres are bridged by two carboxylate parts and one pyo part to form one-dimensional Mg(II) chains with the corner-shared MgO_6_ octahedral units running along the *b*-axis. These one-dimensional chains are further linked by bpdo ligands, resulting in the two-dimensional coordination network (Fig. 2a).

In **2**⊃2DMF, the Mg(II) centre has an octahedral environment with four 1,4-bdc^2−^ oxygen atoms and two bpdo oxygen atoms (Fig. 2b). The corner-shared MgO_6_ octahedral units interconnected by both organic moieties form infinite chains (Supplementary Fig. 2). These chains are further bridged by both bpdo and 1,4-bdc^2−^ ligands, affording a three-dimensional porous coordination framework with one-dimensional channels (Fig. 2c, channel size: 4.5 × 4.1 Å^2^) occupied by guest DMF molecules. The accessible volume of the fully desolvated **2** is ca 35.6%, which was calculated using the PLATON programme (probe radius: 1.2 Å)^30^.

In **3**⊃0.5DMF, the Ca(II) centre has an octahedral environment with four 1,4-bdc^2−^ oxygen atoms and two bpdo oxygen atoms arranged in a *cis* conformation (Fig. 2d). The CaO_6_ octahedral units are interconnected by two carboxylate parts to form one-dimensional Ca(II) chains (Supplementary Fig. 3). These chains are bridged by 1,4-bdc^2−^ ligands, affording two-dimensional layers parallel to the *ac* plane. These layers are further connected by bpdo ligands, forming a three-dimensional porous coordination framework with one-dimensional channels (Fig. 2e, channel size: 3.4 × 3.2 Å^2^) that contain guest DMF molecules. From the calculation using the PLATON programme, the accessible volume of the fully desolvated **3** was found to be ca 21.1%, smaller than that in **2** (ref. 30).

It was confirmed from theoretical and experimental results that the neutral organic bpdo ligand has hard basicity and is a good partner to hard Group II Mg(II) and Ca(II) ions. It should be noted that the NBO charge of oxygen atoms in the bpdo ligand (−0.543 e) is considerably smaller than −1 e, which is the maximum value when only the resonance structures of (I), (II) and (III) contribute to the electronic structure of bpdo. This means that the negative charge on the oxygen atoms of the bpdo ligand may further increase by ionic interactions with cationic Group II metal ions. To clarify this, infrared spectra were measured (Supplementary Figs 4 and 5); these showed that the N–O stretching band of bpdo in **2**⊃2DMF (1,225 cm^−1^) shifts to a lower wavenumber than that of free bpdo ligand (1,242 cm^−1^), suggesting an enhanced single bond character and negative charge accumulation by coordination to Mg(II) ions. Furthermore, the NBO charge of the model structure of **2**, [Mg_4_(bza)_4_(OH)_4_(bpdo)(H_2_O)_8_] (Supplementary Fig. 6), was calculated. As shown in Supplementary Fig. 7, the coordinated bpdo ligand has oxygen atoms with an NBO charge of −0.660 e, 21.5% higher than that of coordination-free bpdo, whereas the NBO charge of the oxygen atoms in the carboxylate ligand, bza^−^, is only changed by −1.1 to 2.1% before and after the coordination, indicating that the hard basicity of the bpdo ligand indeed increases by coordination to cationic Group II Mg(II) ions. The N–O bond of bpdo in the model structure increases in length by further negative charge accumulation on bpdo oxygen atoms (Supplementary Table 1). On the other hand, the corresponding N–O stretching band position in **3**⊃0.5DMF (1,240 cm^−1^) is almost the same as that of the free bpdo ligand, because a Ca(II) ion (effective ion radius: 1.00 Å (ref. 31)) with lower charge density than a Mg(II) ion (effective ion radius: 0.720 Å (ref. 31)) cannot induce further charge accumulation. These results indicate that **2** has more polar channels than **3**, which affects the adsorption properties (vide infra).

### Framework stability

To evaluate the framework stability, thermogravimetry-differential thermal analyses (TG-DTA) and powder X-ray diffraction (XRD) analyses of the desolvated **2** and **3** were performed. **2** was prepared by heating the EtOH-exchanged sample at 373 K under vacuum, whereas **3** was obtained by the direct removal of DMF molecules from **3**⊃0.5DMF at 423 K under vacuum. TG-DTA analysis suggests that **2** and **3** are stable up to 573 and 473 K, respectively (Supplementary Figs 8 and 9). The powder XRD patterns of the desolvated **2** and **3** (Supplementary Figs 10 and 11) show that the original frameworks are stable after the removal of guest molecules.

### Porosity

**Figure 3:**
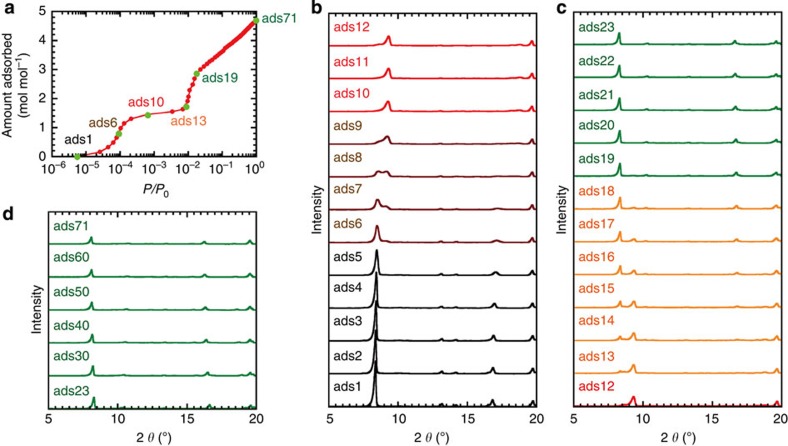
Coincident X-ray diffraction (XRD)/adsorption measurements of **2**. (**a**) CO_2_ adsorption isotherm at 195 K. (**b**–**d**) XRD patterns measured at each point (ads1–ads23, ads30, ads40, ads50, ads60 and ads71).

We first measured the adsorption/desorption isotherms for N_2_ and CO_2_ at low temperature to investigate the fundamental porous properties. **1** shows no CO_2_ adsorption at 195 K (Supplementary Fig. 12), consistent with the fact that **1** has no permanent pores in the X-ray crystal structure. In the PCPs **2** and **3**, CO_2_ and N_2_ gases are adsorbed (Supplementary Figs 13 and 14). The N_2_ adsorption isotherms show type-I curves, indicative of permanent micropores. The Dubinin–Radushkevich plots give the micropore volumes of 0.19 and 0.085 cm^3^ g^−1^ for **2** and **3**, respectively. The CO_2_ adsorption/desorption isotherms in **3** also show type-I curves, whereas **2** shows a stepwise adsorption curve (Supplementary Fig. 14). Coincident infrared/adsorption measurements were performed for various CO_2_ pressures at 195 K to confirm the structural change. If the structural change occurs, an increase in infrared intensity at one point should be observed, with a decrease in infrared intensity at another point. In **3**, the intensities of bands derived from the framework decrease with increasing adsorption (Supplementary Fig. 15), whereas the intensities of some bands increase at first and second adsorption events in **2** (Supplementary Fig. 16), suggesting the structural change of **2** associated with first and second CO_2_ adsorption. To investigate the structural changes of **2** in more detail, coincident XRD/adsorption measurements were conducted. Upon CO_2_ adsorption and desorption, pronounced changes in the XRD patterns were observed (Fig. 3 and Supplementary Fig. 17). The XRD pattern does not change dramatically in the initial CO_2_ adsorption (ads1 to ads5). After ads5, the XRD pattern changes and the structural change finishes at ads10. The structure again begins to change after ads12, and the peak positions in the XRD pattern at ads19 where the second adsorption event is completed are similar to those of the desolvated **2**. Further gradual CO_2_ adsorption after ads19 leads to the slight structural change. In the desorption process, the reverse change in the XRD patterns was observed. Because the crystal structure and the change in the XRD patterns upon CO_2_ adsorption and desorption in **2** are similar to those observed in [Cr(OH)(1,4-bdc)]_*n*_ (MIL-53)^32^, we speculate that the pore contraction and re-expansion occur during CO_2_ adsorption/desorption in **2** (Supplementary Fig. 18).

### Adsorption selectivity

**Figure 4:**
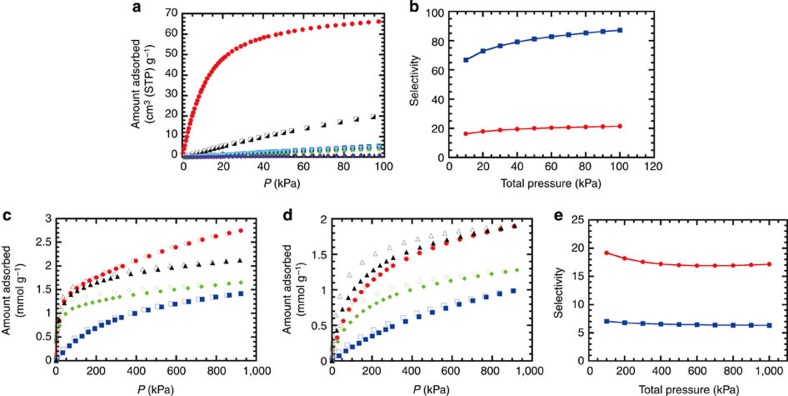
Gas adsorption properties. (**a**) Low-pressure adsorption isotherms for CO_2_ (red circle), CH_4_ (black half-filled square), N_2_ (blue square), O_2_ (green triangle), Ar (sky-blue inverted triangle) and H_2_ (purple rhombus) in **2** at 298 K. Because the amounts adsorbed for N_2_, O_2_ and Ar are almost the same, the symbols overlap. (**b**) Ideal adsorbed solution theory (IAST)-predicted selectivity for CO_2_/CH_4_ (red) and CO_2_/N_2_ (blue) in **2** based on the data measured at 298 K for a bulk gas composition of CO_2_:CH_4_=40:60 (typical composition of biogas) and CO_2_:N_2_=10:90 (typical composition of flue gas). (**c**,**d**) High-pressure total adsorption/desorption isotherms for CO_2_ (red circle), CH_4_ (blue square), C_2_H_6_ (green rhombus), and C_2_H_4_ (black triangle) in **2** (**c**) and **3** (**d**) at 298 K. Adsorption, closed symbols; desorption, open symbols. (**e**) IAST-predicted selectivity for CO_2_/CH_4_ in **2** (red) and **3** (blue) based on the data measured at 298 K for a bulk gas composition of CO_2_:CH_4_=40:60 (typical composition of biogas).

The low-pressure CO_2_, CH_4_, N_2_, O_2_, Ar and H_2_ adsorption isotherms for **2** at 298 K are compared in Fig. 4a. **2** adsorbs very small amounts of N_2_, O_2_, Ar and H_2_, and a small amount of CH_4_ at 298 K. On the other hand, the uptake of CO_2_ at 97 kPa is ~12 times higher than the uptake of N_2_. The effective interaction with polar CO_2_ gas causes a high selectivity for the adsorption of CO_2_ over other gases, which is a prerequisite for the application of a separation material. The adsorption of gas mixtures in porous materials can be reliably estimated from the ideal adsorbed solution theory (IAST), which is a precise method used to describe gas-mixture adsorption in representative zeolites and PCPs from the experimental single-component gas adsorption isotherms^33^. The predicted adsorption selectivities for CO_2_/CH_4_ and CO_2_/N_2_ for a bulk gas composition of CO_2_:CH_4_=40:60 (typical composition of biogas) and CO_2_:N_2_=10:90 (typical composition of flue gas) are 21–16 and 87–67, respectively (Fig. 4b), high enough (>8) for the potential feasibility of the practical procedure^34^. The IAST selectivity for other compositions (including typical compositions in the air or ternary mixture) is also high (Supplementary Figs 19–24). In addition, high-pressure adsorption measurements were performed at 298 K (Fig. 4c,d), which confirmed the favourable adsorption of CO_2_ and C2 hydrocarbons (C_2_H_6_ and C_2_H_4_) relative to CH_4_ (see Supplementary Figs 25 and 26). The IAST selectivity of CO_2_ relative to CH_4_ is still larger than 8 for **2** and slightly smaller than 8 for **3** (Fig. 4e), indicating the practical implementability for biogas purification (vide infra).

Recently, Snurr *et al*. have used five adsorbent evaluation criteria from the chemical engineering literature for the potential use of PCPs/MOFs in CO_2_ separation processes^35^. We focus on flue gas separation, which is currently an important research area. The typical composition of flue gas, that is, the CO_2_/N_2_ ratio, is assumed to be 10:90 and the adsorption/desorption pressures are set to 100/10 kPa, respectively. Based on this condition, **2** was evaluated and compared not only with other PCPs/MOFs but also with commercially available inorganic and organic adsorbents (see Supplementary Table 2). As shown in Supplementary Table 2, **2** has high working capacity (or regenerability) and selectivity under these conditions, resulting in a high sorbent selection parameter (*S*) that combines the working capacity and the adsorption selectivity. The *S* value obtained (314) is considerably higher than those of zeolites (163 for zeolite 5A and 128 for zeolite 13X) and one of the best among the PCPs/MOFs.

The regenerability of the CO_2_ adsorption process in **2** was measured at 298 K (Supplementary Fig. 27). Significantly, the CO_2_ adsorption ability of **2** is maintained over repeated cycling, and the material can be regenerated by short vacuum processing without additional heating, in contrast to zeolites, which require high temperatures for complete regeneration (Supplementary Figs 28 and 29). The isosteric heat of CO_2_ adsorption, *Q*_st_, shows values 32–36 kJ mol^−1^ (zero-coverage *Q*_st_=34.9 kJ mol^−1^, Supplementary Fig. 30), which are smaller than those of amine-functionalized PCPs and zeolites^36^,^37^. Such a moderate *Q*_st_ value is a strong advantage for the implementation of low-energy regeneration for CO_2_ separation.

**Figure 5:**
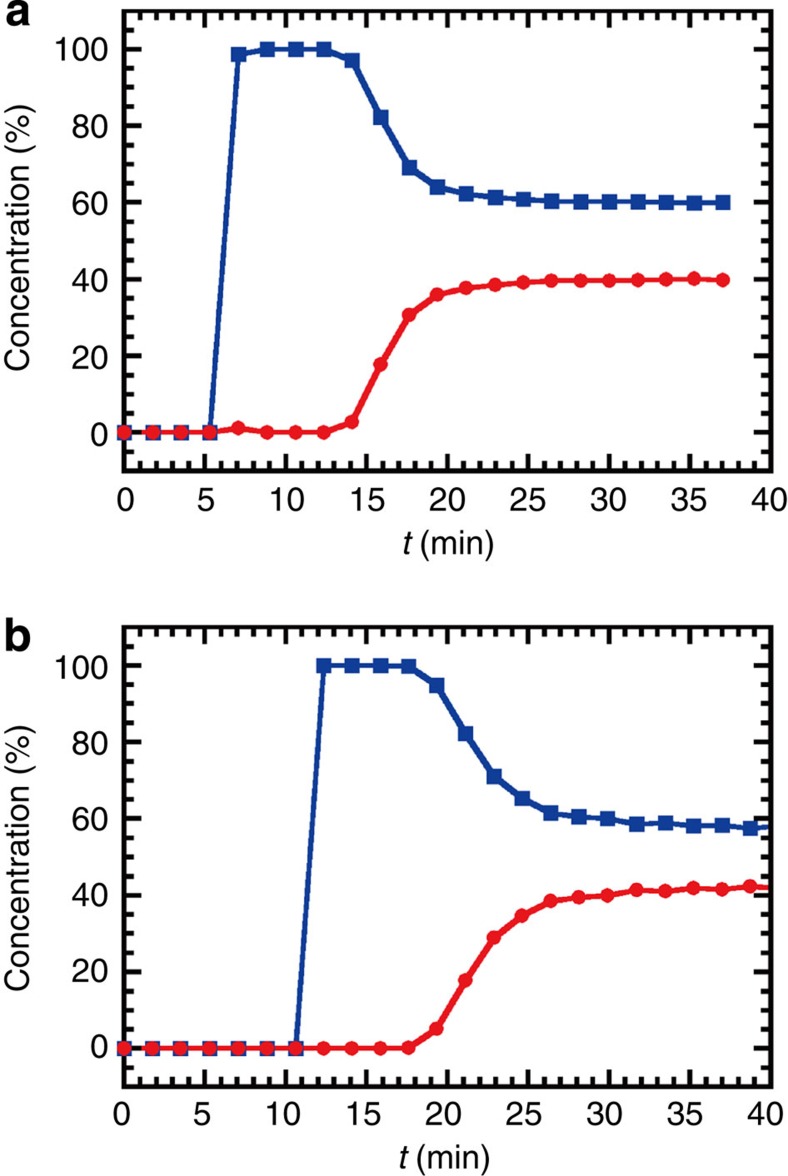
Gas separation performance. (**a**,**b**) Breakthrough curves for a CO_2_/CH_4_ mixture (40:60 (vol)) for **2** (**a**) and **3** (**b**). The red circle is CO_2_ and the blue square is CH_4_. These were measured at 298 K, the total pressure was 0.8 MPa and the space velocity was 3 min^−1^.

To evaluate the gas separation ability of adsorbents, it is important to study not only the single-gas adsorption properties and IAST simulation under equilibrium conditions, but also mixed gases under flowing (kinetic) conditions. Thus, we conducted a breakthrough experiment, which is a typical way of evaluating the gas separation ability of adsorbents under flowing gas conditions that are related to the pressure swing adsorption (PSA) process. We measured the gas separation properties of **2** and **3** for the CO_2_:CH_4_=40:60 mixture at 298 K and under a total pressure of 0.8 MPa. The separation of CO_2_ from CO_2_/CH_4_ mixtures in biogas has become increasingly important in recent times. The PSA process is a well-known technology and the selective binding of the target gas and gas adsorption kinetics are important factors in the design of adsorbent materials. PSA typically runs at ambient temperature and under a total pressure of 0.4–0.8 MPa, and the cycle time is several minutes. As shown in Fig. 5, the gas detected first by gas chromatography was CH_4_ only, with no detection of CO_2_, indicating high selectivity for CO_2_ over CH_4_ under flowing conditions. After a few minutes, the process reached the breakpoint, and then returned to the original gas ratio. It therefore seems that **2** and **3** have favourable characteristics for CO_2_ capture, yielding good separation behaviour.

Although high water stability and hydrophobicity are also one of important factors in using gas separation materials under practical conditions, syntheses of PCPs/MOFs showing high CO_2_ selectivity and no degradation of frameworks under humid environments have been just started^38^,^39^,^40^. Here we tested the stability of frameworks after exposure of **2** and **3** to water vapour. **2** adsorbs a large amount of H_2_O (Supplementary Fig. 31), and the adsorbed amount of CO_2_ in **2** after the H_2_O adsorption shows a decrease of 65% compared with the as-synthesized **2** (Supplementary Fig. 32), suggesting that **2** is not stable in humid conditions. On the other hand, a few amount of H_2_O is adsorbed to **3** at 37% relative humidity, and the adsorbed amount of CO_2_ is unchanged after the H_2_O adsorption experiment (Supplementary Fig. 33), indicating higher water stability of **3** than **2**. However, it is necessary to further improve the stability to water and evaluate gas separation properties under humid conditions.

## Discussion

To our knowledge, almost all PCPs with Group II Mg(II) and Ca(II) ions contain only anionic organic ligands; there are few examples including bridging neutral organic ligands. **2** and **3** are the uncommon examples of Group II Mg(II) and Ca(II) PCPs including bridging neutral organic ligands. Note that we can use a variety of neutral organic ligands with pyo moieties, as shown in Supplementary Fig. 34a. In contrast to a monodentate neutral pyridine ligand, even the most simple pyo ligand is capable of bridging metal cations because the coordinated oxygen has two lone-pair electrons. Furthermore, pyo ligands with carboxylate substituents (for example, pyridine-4-carboxylate-*N*-oxide; see Supplementary Fig. 34b) are also useful building blocks for the construction of Group II Mg(II) and Ca(II) PCPs. These considerations strongly suggest that our proposed strategy to use pyo-type ligands will be quite effective for the diversification of Group II Mg(II) and Ca(II) PCP frameworks. We can expect the pyo-type ligands to also be good partners to monovalent Group I alkali Na(I) and K(I) ions. Although successful studies on syntheses of coordination polymers with Group I alkali metal ions have recently been performed using the neutral cyclodextrin and polyether ligands^22^,^41^,^42^,^43^, there are still few PCPs containing Group I alkali Na(I) and K(I) ions. Their hard acidity impedes the coordination of neutral organic ligands, and the monovalence restricts the number of anionic multicarboxylate ligands (such as 1,4-bdc^2−^ and 1,3,5-benzenetricarboxylate) coordinating to one Group I alkali Na(I) and K(I) ion, which causes difficulty in the formation of porous frameworks. Because of their neutrality and hard basicity, the pyo-type ligands are good candidates to resolve this problem. Therefore, we conclude that the structural diversification of PCPs with Group I Na(I) and K(I) ions and Group II Mg(II) and Ca(II) ions that are more abundant on the Earth and more biofriendly than commonly used transition metal ions would be realized by utilizing pyo-type ligands.

In conclusion, we succeeded in finding a new partner, charge-polarized neutral ‘bpdo’ ligand, for Group II Mg(II) and Ca(II) ions in order to realize the structural diversification of Mg(II) and Ca(II) PCPs. The bpdo ligand has hard basicity, derived from its polarized structure, which causes the neutral bpdo to bridge hard Mg(II) and Ca(II) ions. The obtained Mg(II) and Ca(II) PCPs with neutral bpdo ligands show sufficient framework stability and good gas separation ability. Our finding opens up the science and engineering of PCPs with cheap and biocompatible Group I and Group II metal ions.

## Methods

### Materials

All commercially available starting materials were purchased from Wako Pure Chemical Industries, Ltd., and used as received. The solvents for the syntheses were used without further purification. bpdo was synthesized according to the literature^44^.

### Synthesis of [Mg_2_(AcO)_4_(bpdo)]_*n*_ (**1**)

A DMF solution (5 ml) of Mg(AcO)_2_·6H_2_O (107 mg, 0.50 mmol) and bpdo (94 mg, 0.50 mmol) was heated at 423 K for 6 h. The obtained colourless microcrystals were filtered, dispersed in a DMF solution (20 ml) and heated at 373 K for 10 min. The obtained colourless microcrystals of [Mg_2_(AcO)_4_(bpdo)]_*n*_ (**1**) were filtered, washed with MeOH and dried at 298 K under vacuum for 2 h. Yield: 97 mg, 82%. Elemental analysis: Calcd for **1** (C_18_H_20_Mg_2_N_2_O_10_): C=45.71; H=4.26; N=5.92. Found: C=44.10; H=4.32; N=5.44%.

### Synthesis of {[Mg_2_(1,4-bdc)_2_(bpdo)]·2DMF}_*n*_ (2⊃2DMF)

A DMF solution (5 ml) of MgCl_2_·6H_2_O (203 mg, 1.00 mmol), 1,4-H_2_bdc (166 mg, 1.0 mmol) and bpdo (94 mg, 0.50 mmol) was heated at 423 K for 24 h. The obtained colourless microcrystals of {[Mg_2_(1,4-bdc)_2_(bpdo)]·2DMF}_*n*_ (**2**⊃2DMF) were filtered, washed with DMF and dried at 298 K under vacuum for 2 h. Yield: 292 mg, 41%. Elemental analysis: Calcd for **2**⊃1.8DMF·0.6H_2_O (C_31.4_H_29.8_Mg_2_N_3.8_O_12.4_): C=53.31; H=4.25; N=7.52. Found: C=52.69; H=4.43; N=7.63%.

### Synthesis of {[Ca(1,4-bdc)(bpdo)]·0.5DMF}_*n*_ (3⊃0.5DMF)

A DMF solution (40 ml) of Ca(NO_3_)_2_·4H_2_O (708 mg, 3.00 mmol), 1,4-H_2_bdc (498 mg, 3.00 mmol) and bpdo (1.13 g, 6.00 mmol) was heated at 423 K for 48 h. The obtained colourless microcrystals of {[Ca(1,4-bdc)(bpdo)]·0.5DMF}_*n*_ (**3**⊃0.5DMF) were filtered, washed with DMF and dried at 298 K under vacuum for 2 h. Yield: 1.20 g, 97%. Elemental analysis: Calcd for **3**⊃0.3DMF (C_18.9_H_4.1_CaN_2.3_O_6.3_): C=54.79; H=3.43; N=7.78. Found: C=53.44; H=3.68; N=7.73%.

### Physical measurements

Elemental analyses (C, H and N) were performed using a Yanaco CHN corder MT-6. The infrared spectra were recorded using KBr disks on a Thermo Nicolet 6700 FT-IR spectrometer with a resolution of 4 cm^−1^. TG-DTA analysis was performed using a Rigaku ThermoPlus2/TG-DTA8129 over the temperature range r.t.–500 °C under a N_2_ flow at a heating rate of 10 °C min^−1^. Powder XRD data of microcrystals were collected using a Rigaku RINT-Ultima III diffractometer employing Cu Kα radiation. The adsorption and desorption isotherms for CO_2_ (195, 288 and 298 K), CH_4_ (298 K), N_2_ (77 and 298 K), O_2_ (298 K), Ar (298 K) and H_2_ (298 K) were recorded on a BELSORP-max volumetric adsorption instrument (BEL Japan, Inc., Supplementary Table 3). The adsorption isotherm measurements for H_2_O at 298 K were performed using a BELSORP-aqua volumetric adsorption instrument (BEL Japan, Inc.). The high-pressure adsorption and desorption isotherms for CO_2_, CH_4_, C_2_H_4_ and C_2_H_6_ (298 K) were measured with a BELSORP-HP volumetric adsorption equipment (BEL Japan, Inc., Supplementary Tables 4 and 5). We estimated the total adsorbed amount using the simple equation (1)^45^:




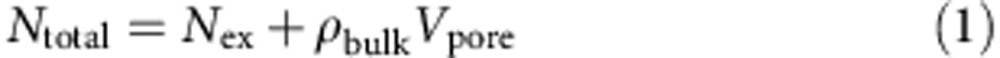




where *N*_total_ is the total adsorbed amount, *N*_ex_ is the surface excess amount, *ρ*_bulk_ is the bulk density of the gases and *V*_pore_ is the pore volume of the PCPs. Total adsorption was calculated using NIST Thermochemical Properties of Fluid Systems: CO_2_, CH_4_, C_2_H_4_ and C_2_H_6_ densities between 0 and 2,000 kPa were fitted using a sixth-order polynomial, then multiplied by the pore volume of each material^46^. The IAST of Prausnitz and Myers was used to estimate the composition of the adsorbed phase from pure component isotherm data^33^. Experimental isotherm data were fitted to the single-site or dual-site Langmuir–Freundlich model (Supplementary Table 6). Selectivities were calculated using the following expression (2):




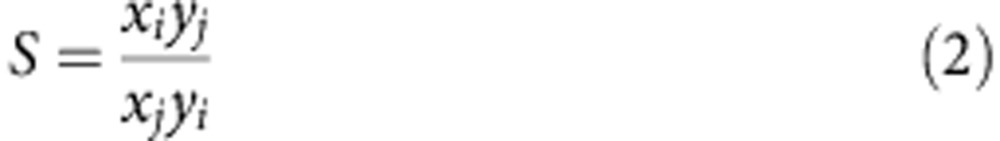




where *x*_*i*_ is the mole fraction of component *i* in the adsorbed phase and *y*_*i*_ is the mole fraction of component *i* in the bulk. The coverage-dependent isosteric heat of adsorption was evaluated by first fitting the temperature-dependent isotherm data (288 and 298 K, Supplementary Figs 35 and 36) to a virial-type expression^47^, which can be written as follows:




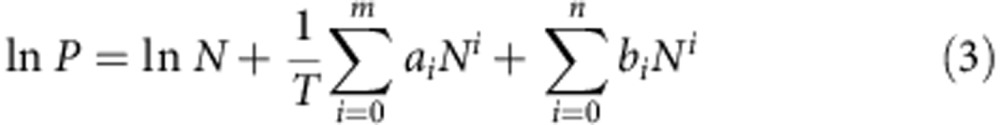




where *P* is the pressure, *N* is the quantity of CO_2_ adsorbed, *T* is the temperature, *a*_*i*_ and *b*_*i*_ are virial coefficients, and *m* and *n* are the number of virial coefficients required for adequate fitting of the isotherms. Then, the isosteric heat of adsorption was evaluated using the following expression (4):




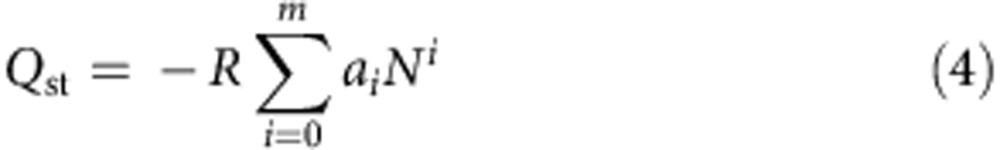




where *R* is the universal gas constant. The zero-coverage isosteric heat of adsorption is given by:




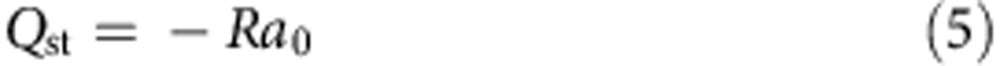




Repeated adsorption measurements were performed at 298 K using the BELSORP-max. The samples after the adsorption measurement were degassed at 298 K under vacuum for 1 min and used for the next measurement. Molecular Sieves 13X was used as a reference sample. Coincident infrared/adsorption measurements were carried out at 195 K using a JASCO model VIR-200 Fourier transform infrared spectrometer connected to a BELSORP-18PLUS volumetric adsorption instrument (BEL Japan, Inc.) with neat samples. Those apparatuses were synchronized with each other and each infrared spectrum was obtained at each equilibrium point of the adsorption/desorption isotherms. The initial infrared spectrum before CO_2_ dosage was used as the reference for obtaining the background-subtracted spectra. Coincident XRD/adsorption measurements were performed at 195 K using a Rigaku UltimaIV with Cu Kα radiation connected to a BELSORP-18PLUS. Those apparatuses were synchronized with each other and each powder XRD pattern was obtained at each equilibrium point of the adsorption/desorption isotherms. Breakthrough curve measurements were performed using a hand-made gas flowing system. The sample cell was filled with sample powders, and the temperature of the cell was controlled by a refrigerant circulating system. The gas ratio was CO_2_:CH_4_=40:60 (vol) and the measurements were executed at 0.80 MPa of total pressure at 298 K with a space velocity of 3 min^−1^. The pressure of CO_2_ was 0.32 MPa.

### Computational details

Geometry optimizations of organic ligands and a model of **2** were carried out using density functional theory with the B3LYP functional^48^,^49^,^50^,^51^. We constructed a finite model based on the X-ray crystal structure of **2**, where four oxygen atoms of bpdo and eight oxygen atoms of bridging carboxylate ligands were replaced by hydroxide ions and water molecules to retain the coordinative environments of Mg, as shown Supplementary Fig. 6. In an optimization process, we fixed the four Mg atoms to the relative positions observed in the crystal structure, whereas all geometries of organic ligands were optimized. The 6-31G(d, p) basis sets were employed for all atoms, where one set of diffuse functions was added to the O atoms in carboxylate ligands, bpdo ligands and hydroxide ions. All geometry optimizations, NBO analysis and evaluation of NBO atomic charges were performed using the Gaussian 09 package^52^.

### Crystal structure determination

Single-crystal XRD measurements of **1**, **2**⊃2DMF and **3**⊃0.5DMF were performed using a Rigaku RAXIS–RAPID imaging plate diffractometer with graphite-monochromated Mo Kα radiation (*λ*=0.71075 Å). The data were corrected for Lorentz and polarization effects. The structures were solved using direct methods (SIR2004 (**1**) and SHELXS-97 (**2**⊃2DMF and **3**⊃0.5DMF)) and expanded using Fourier techniques^53^,^54^. All nonhydrogen atoms were refined anisotropically. All hydrogen atoms were refined using the riding model. The refinements were carried out using full-matrix least-squares techniques on *F*^2^ using SHELXL-97 (**1** and **2**⊃2DMF) and SHELXL-2014 (**3**⊃0.5DMF)^54^ (Supplementary Table 7 and Supplementary Data 1–3). For **3**⊃0.5DMF, the thermal displacement parameters for one of the disordered bpdo molecules were restrained using the *DELU* and *SIMU* commands, and the disordered DMF solvent was refined using the *DFIX*, *DANG*, *FLAT* and *EADP* commands. For **1** and **2**⊃2DMF, all calculations were performed using the CrystalStructure software package^55^. For **3**⊃0.5DMF, all calculations were performed using the WinGX software package^56^.

## Author contributions

S.-i.N. designed this study, interpreted the results and wrote the paper. S.-i.N. and J.M. performed the experiments. Y.H. carried out the calculations. R.M., H.S. and S.K. performed the coincident infrared/adsorption and XRD/adsorption measurements. K.S. carried out the structural analysis. Y.I. performed the high-pressure adsorption and breakthrough curve measurements. All authors discussed the results and commented on the manuscript.

## Additional information

**How to cite this article:** Noro, S.-i. *et al*. Porous coordination polymers with ubiquitous and biocompatible metals and a neutral bridging ligand. *Nat. Commun.* 6:5851 doi: 10.1038/ncomms6851 (2015).

**Accession codes**: The X-ray crystallographic coordinates for structures reported in this Article have been deposited at the Cambridge Crystallographic Data Centre (CCDC), under deposition numbers 997086 to 997088. These data can be obtained free of charge from the Cambridge Crystallographic Data Centre via www.ccdc.cam.ac.uk/data_request/cif

## Supplementary Material

Supplementary Figures, Supplementary Tables, and Supplementary References. Supplementary Figures 1-36, Supplementary Tables 1-7, and Supplementary References

Supplementary Data 1 Crystallographic Information File for compound 1.

Supplementary Data 2 Crystallographic Information File for compound 2.

Supplementary Data 3 Crystallographic Information File for compound 3.
