# Supplementary material for: Porous coordination polymers with ubiquitous and biocompatible metals and a neutral bridging ligand
Source: Nat Commun. 2015 Jan 16;6:5851. doi: 10.1038/ncomms6851 (PMC4309420; doi:10.1038/ncomms6851)
Supplement: Supplementary Figures, Supplementary Tables, and Supplementary References. — Supplementary Figures 1-36, Supplementary Tables 1-7, and Supplementary References [file ncomms6851-s1.pdf]

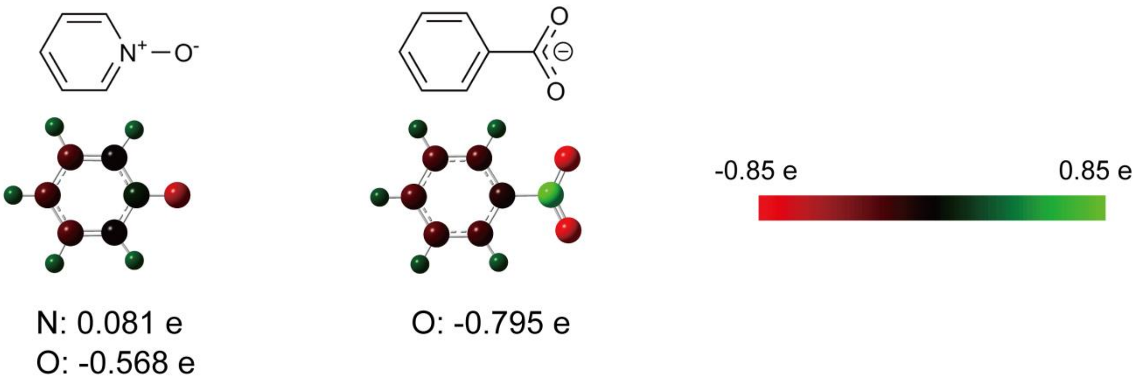

Supplementary Figure 1. Natural bond orbital charges for selected atoms in organic pyridine-*N*-oxide and benzoate ligands.

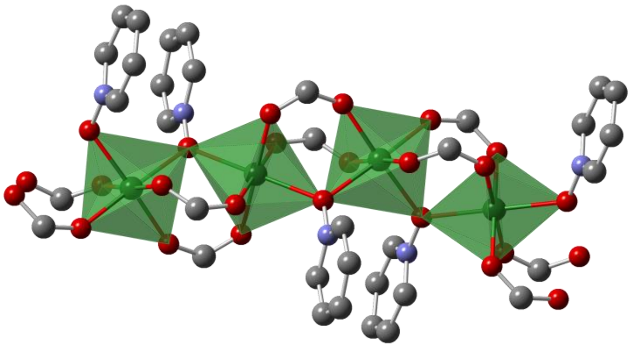

Supplementary Figure 2. The infinite chain of corner-shared MgO_6 octahedral units interconnected by organic moieties in 2⊃2DMF (DMF = *N*,*N*-dimethylformamide).

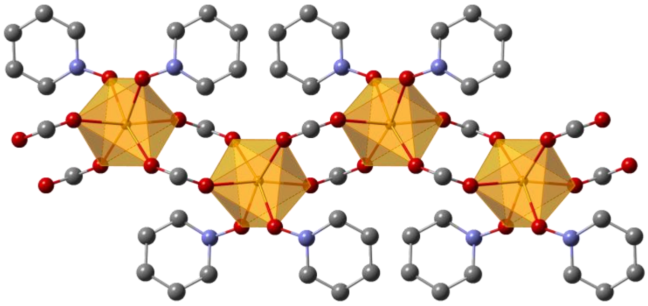

Supplementary Figure 3. One-dimensional chain structure in 3⊃0.5DMF (DMF = *N*,*N*-dimethylformamide).

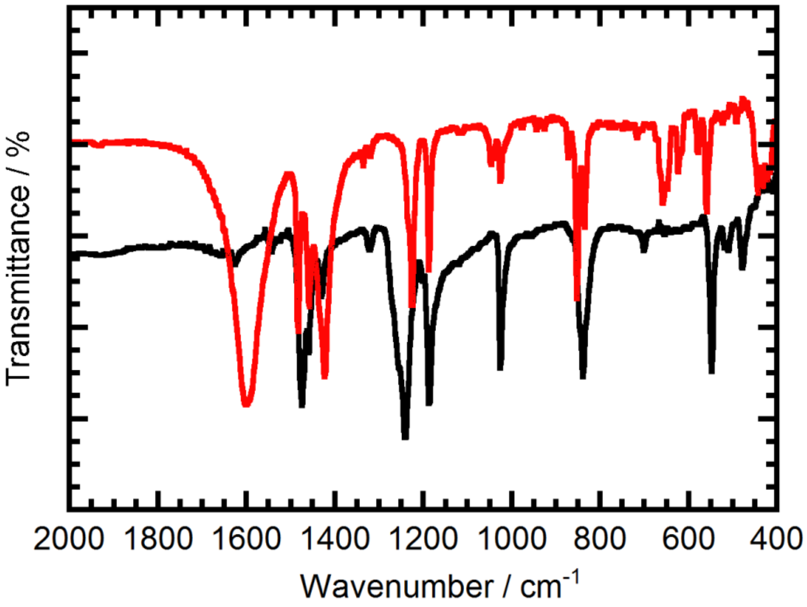

Supplementary Figure 4. Infrared spectra of 4,4′-bipyridine-*N*,*N*′-dioxide (black) and 1 (red).

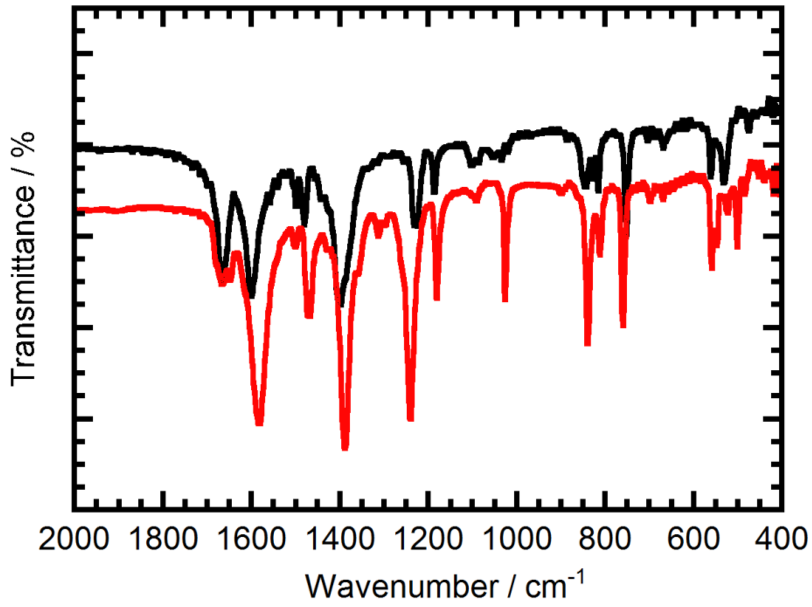

Supplementary Figure 5. Infrared spectra of 2⊃2DMF (black) and 3⊃0.5DMF (red) (DMF = *N*,*N*-dimethylformamide).

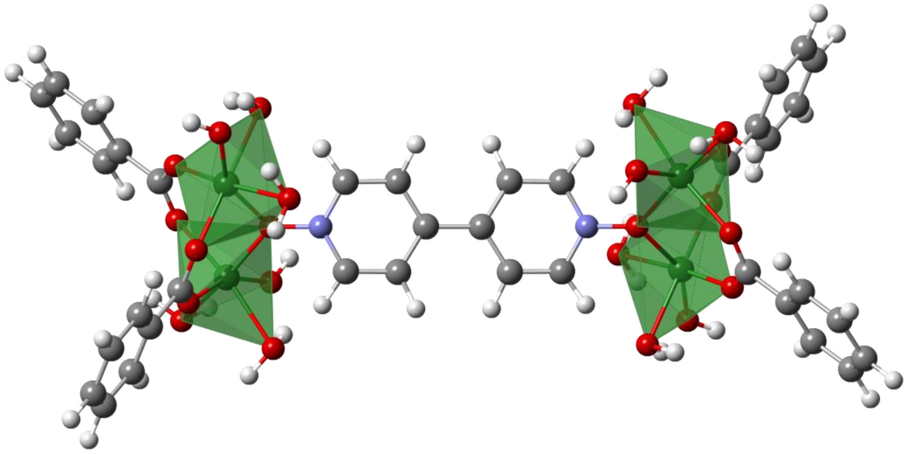

Supplementary Figure 6. Optimized finite model structure, [Mg_2(bza)$_4$(OH)$_4$(bpdo)(H_2O)$_8$], of 2 (bza$^-$ = benzoate and bpdo = 4,4′-bipyridine-*N*,*N*′-dioxide). Green, gray, blue, red, and white atoms indicate magnesium, carbon, nitrogen, oxygen, and hydrogen, respectively.

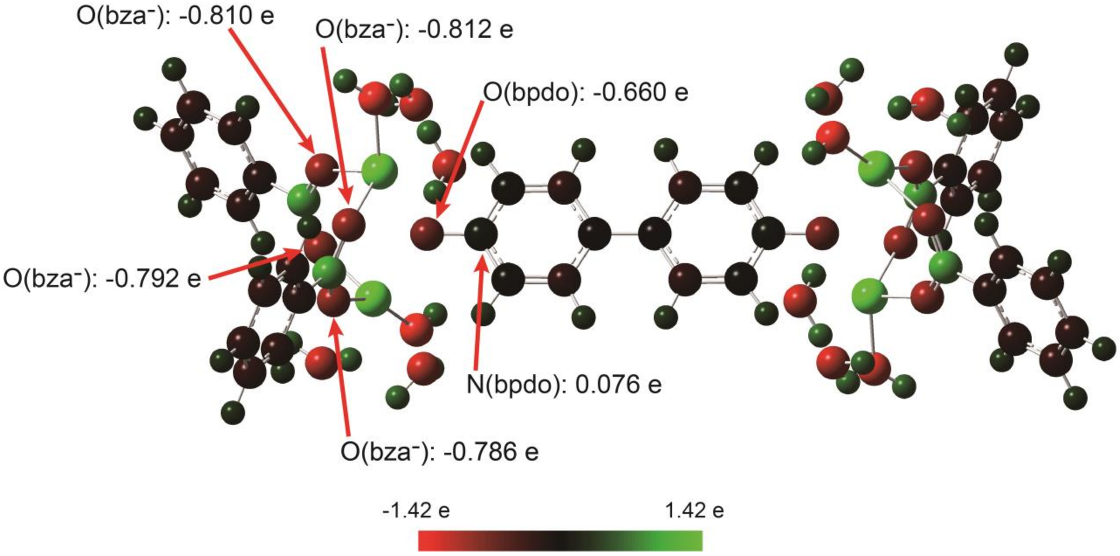

Supplementary Figure 7. Natural bond orbital charges for selected atoms in the model structure [Mg_2(bza)$_4$(OH)$_4$(bpdo)(H_2O)$_8$] (bza^- = benzoate and bpdo = 4,4′-bipyridine-*N*,*N*′-dioxide).

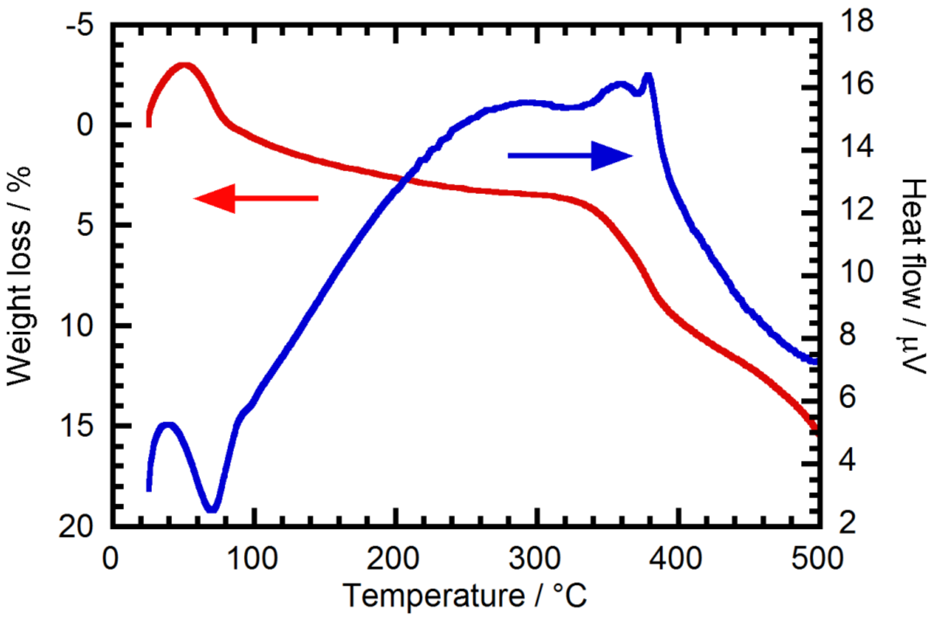

Supplementary Figure 8. Thermogravimetry-differential thermal analysis (TG-DTA) curves of 2⊃x(guest). The desolvated **2** quickly adsorbs atmospheric H_2O after an exposure to air and N_2 flowing through the electric furnace of TG-DTA, which causes the weight loss between r.t. and 573 K.

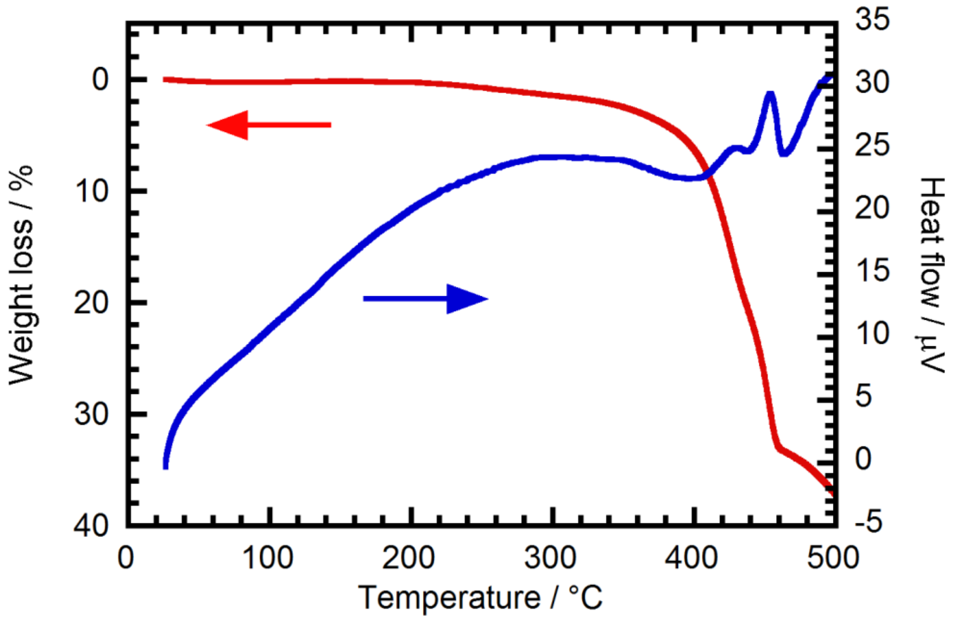

Supplementary Figure 9. Thermogravimetry-differential thermal analysis curves of 3.

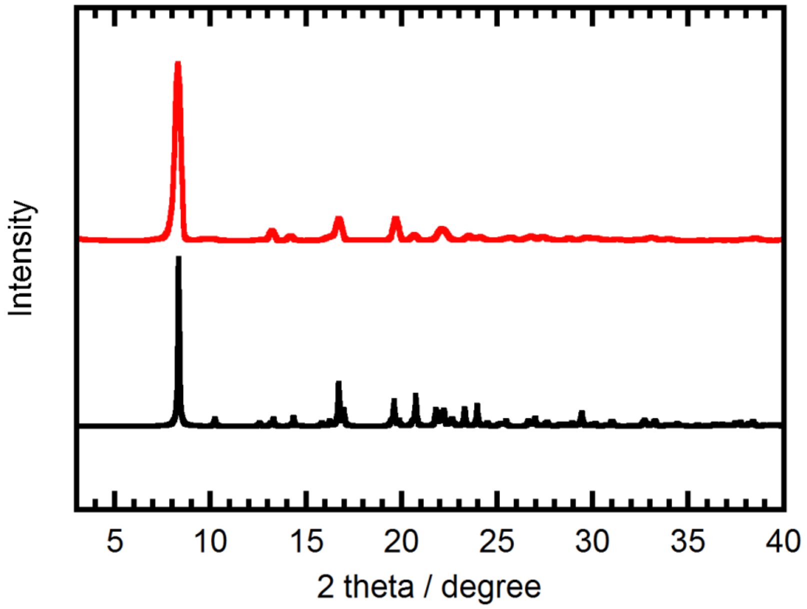

Supplementary Figure 10. Simulated (black) and observed (473 K under vacuum, red) powder X-ray diffraction patterns of 2.

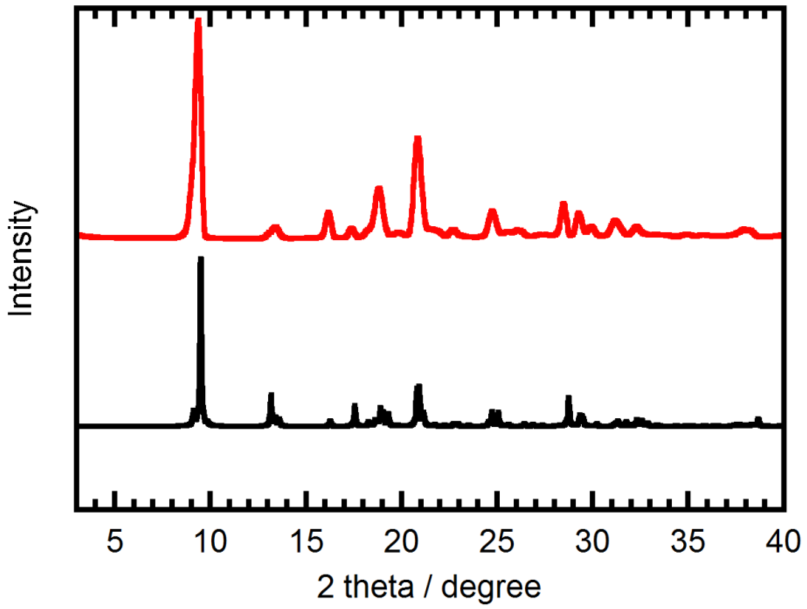

Supplementary Figure 11. Simulated (black) and observed (473 K under vacuum, red) powder X-ray diffraction patterns of 3.

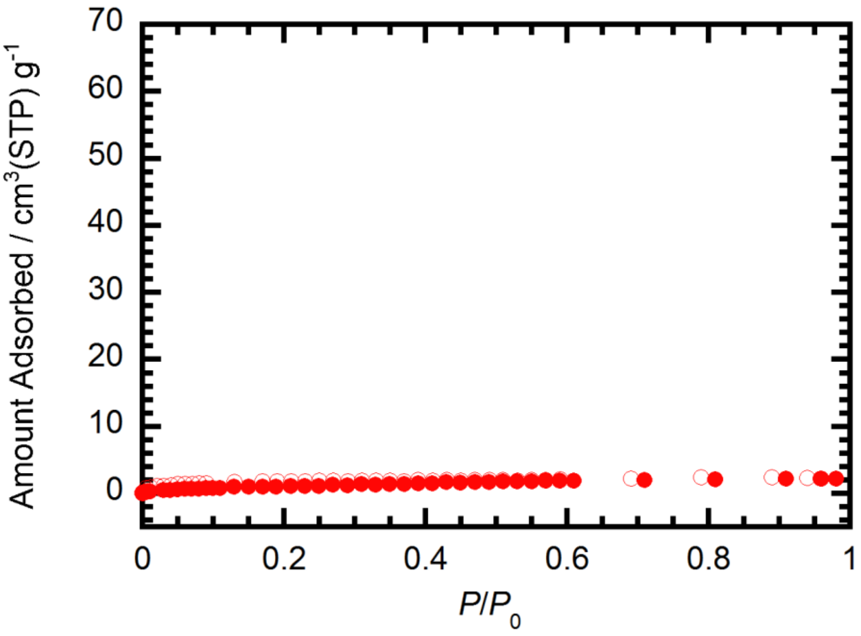

Supplementary Figure 12. CO_2 adsorption/desorption isotherms for 1 at 195 K. Adsorption, closed symbols; desorption, open symbols.

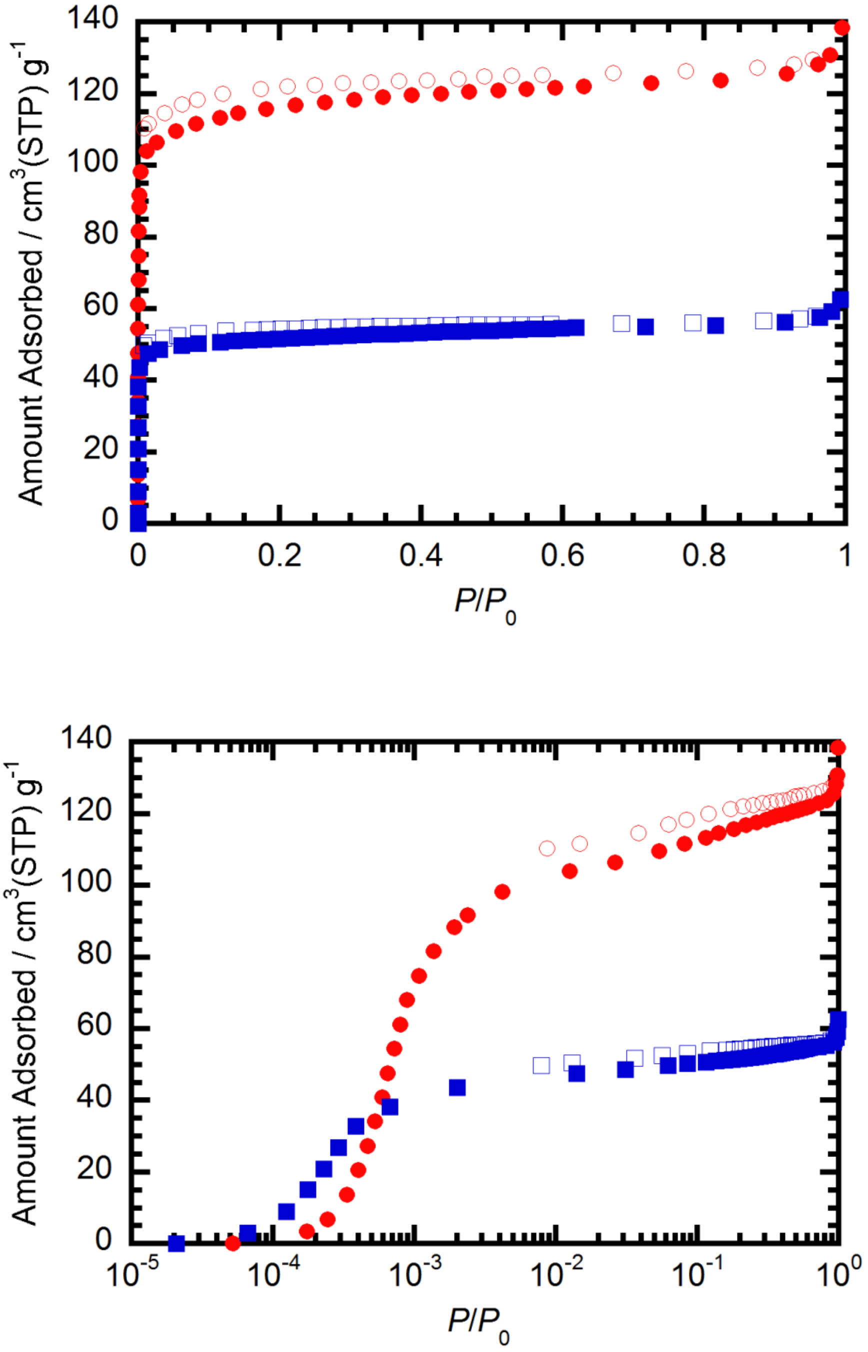

Supplementary Figure 13. N_2 adsorption/desorption isotherms for 2 (red) and 3 (blue) at 77 K. Adsorption, closed symbols; desorption, open symbols; top, standard coordinates; bottom, semi-log plot.

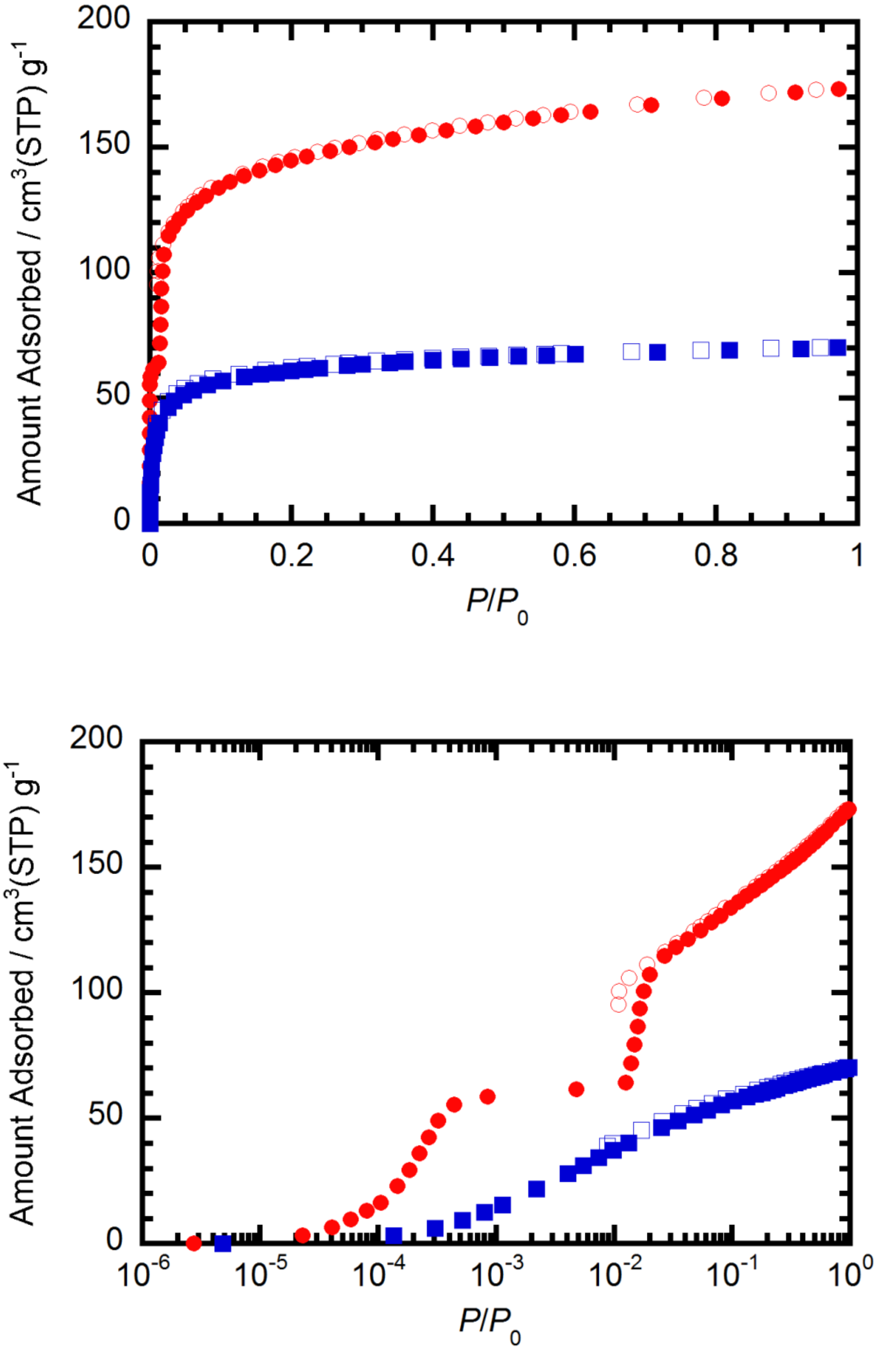

Supplementary Figure 14. CO_2 adsorption/desorption isotherms for 2 (red) and 3 (blue) at 195 K. Adsorption, closed symbols; desorption, open symbols; top, standard coordinates; bottom, semi-log plot.

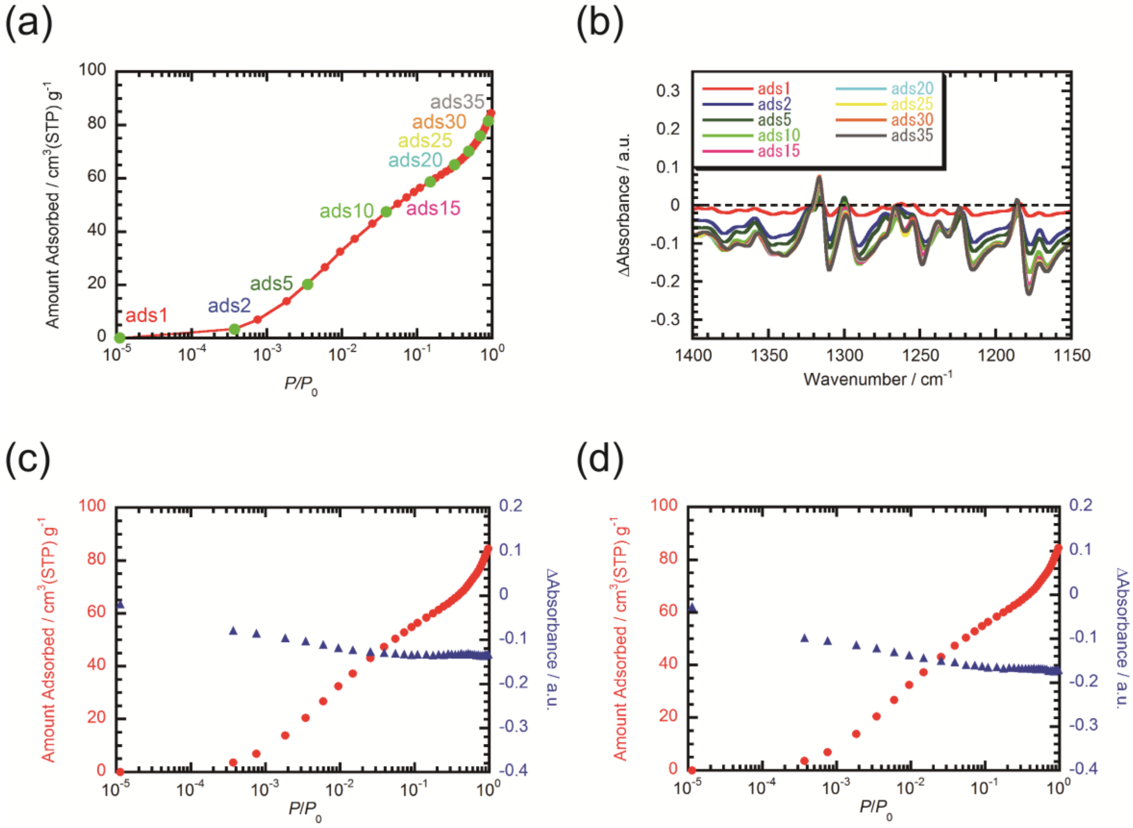

Supplementary Figure 15. Coincident infrared (IR)/adsorption measurements in 3. (a) CO_2 adsorption isotherm at 195 K. (b) Differential IR spectra at each point (ads1 to ads35). (c and d) Amount adsorbed and difference in IR peak intensity as a function of CO_2 relative pressure, where the data were taken at (c) 1339 and (d) 1215 cm^{-1}.

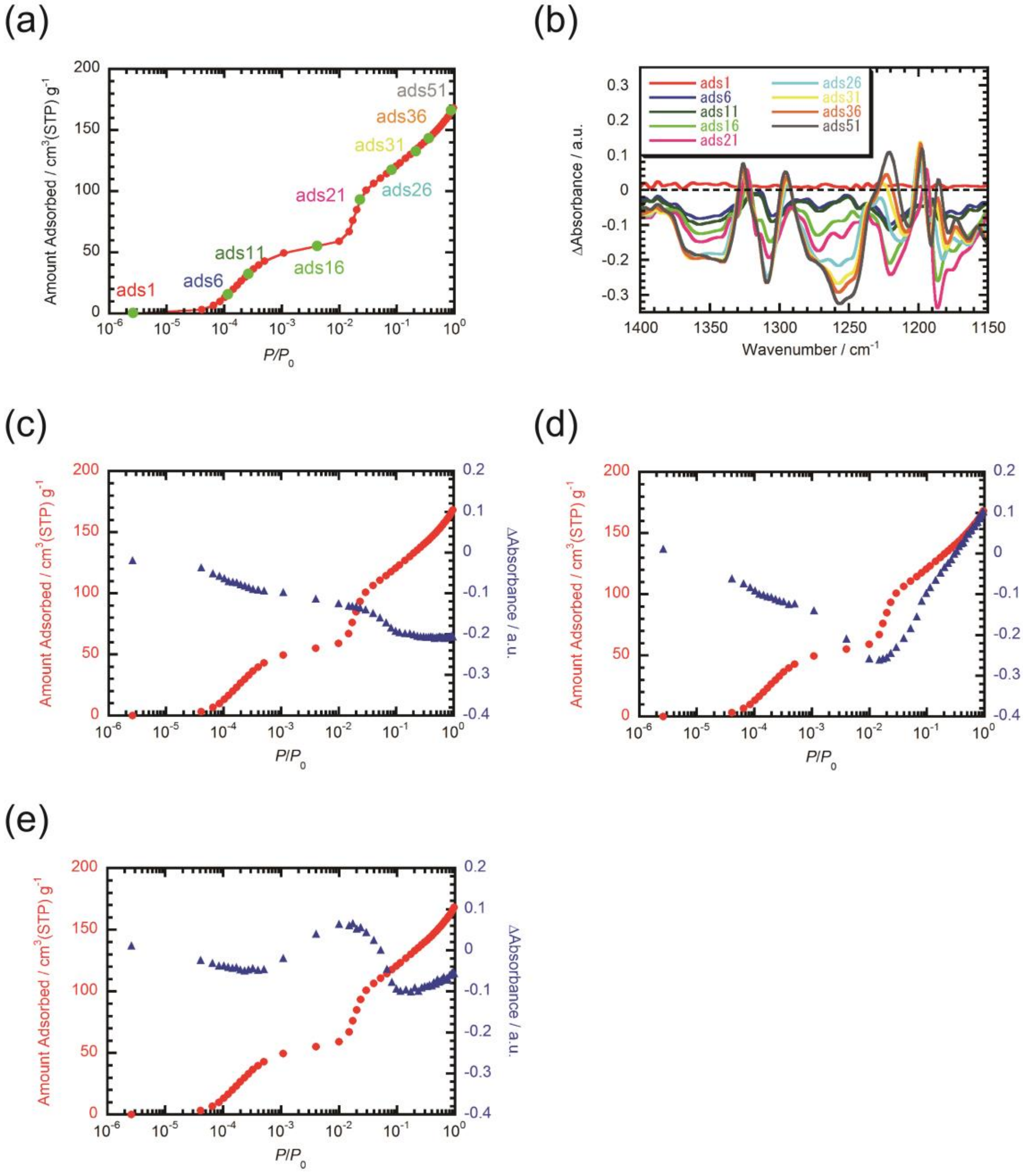

Supplementary Figure 16. Coincident infrared (IR)/adsorption measurements in 2. (a) CO_2 adsorption isotherm at 195 K. (b) Differential IR spectra at each point (ads1 to ads51). (c, d, and e) Amount adsorbed and difference in IR peak intensity as a function of CO_2 relative pressure, where the data were taken at (c) 1341, (d) 1220, and (e) 1194 cm^{-1}.

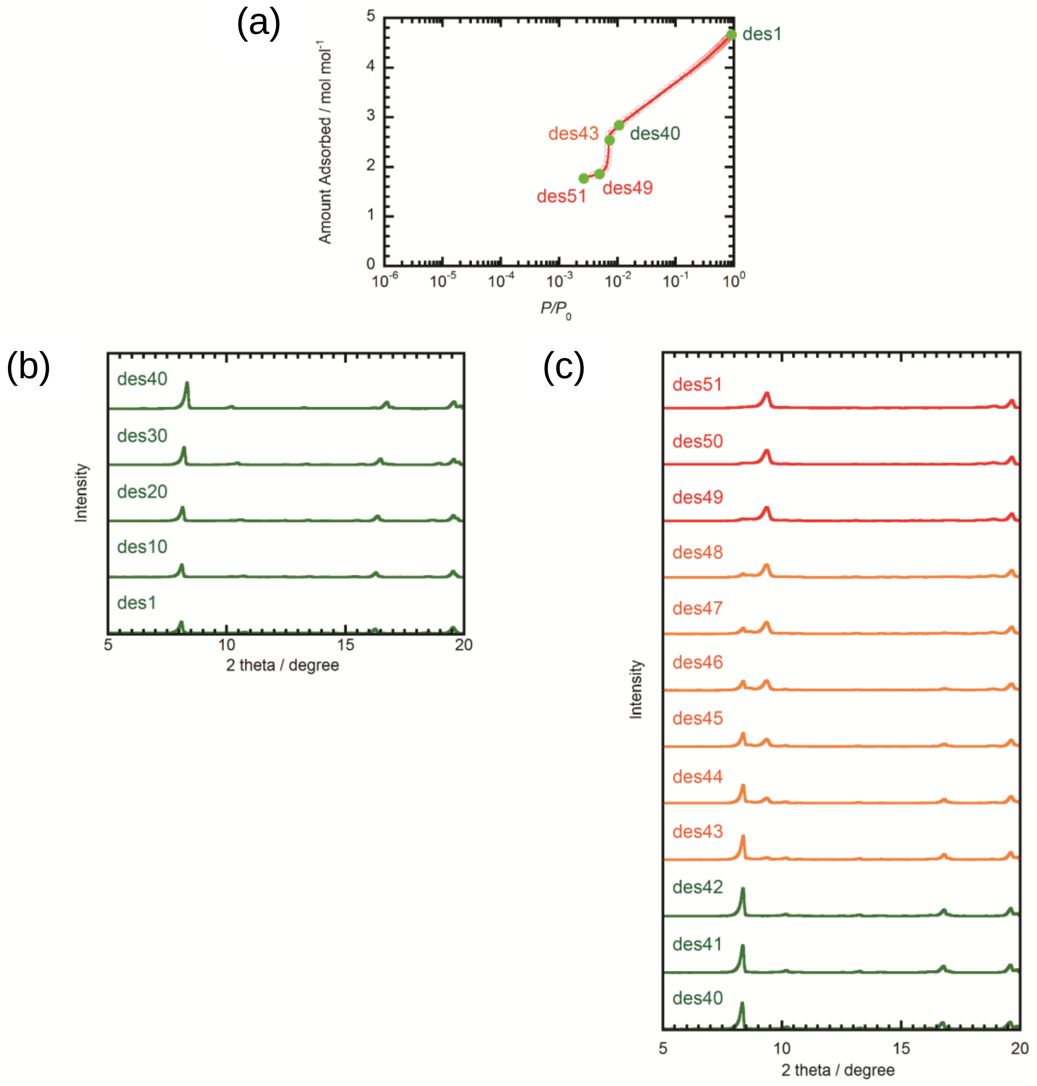

Supplementary Figure 17. Coincident X-ray diffraction (XRD)/desorption measurements of 2. (a) CO_2 desorption isotherm at 195 K and (b and c) XRD patterns measured at each point (des1, des10, des20, des30, and des40~ads51).

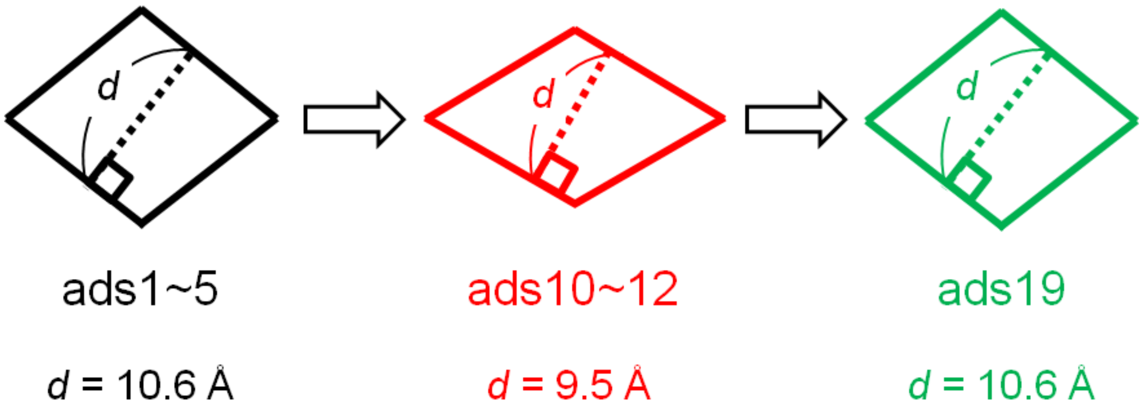

Supplementary Figure 18. Schematic representation of the pore contraction and re-expansion during CO_2 adsorption in 2. The (11-1) reflection observed at the lowest angle corresponds to the distance *d*. This (11-1) reflection shifted to a higher angle during ads5~10, indicating the pore contraction. Further CO_2 adsorption (ads12~19) led to the shift of the (11-1) reflection to the original position, suggesting the re-expansion.

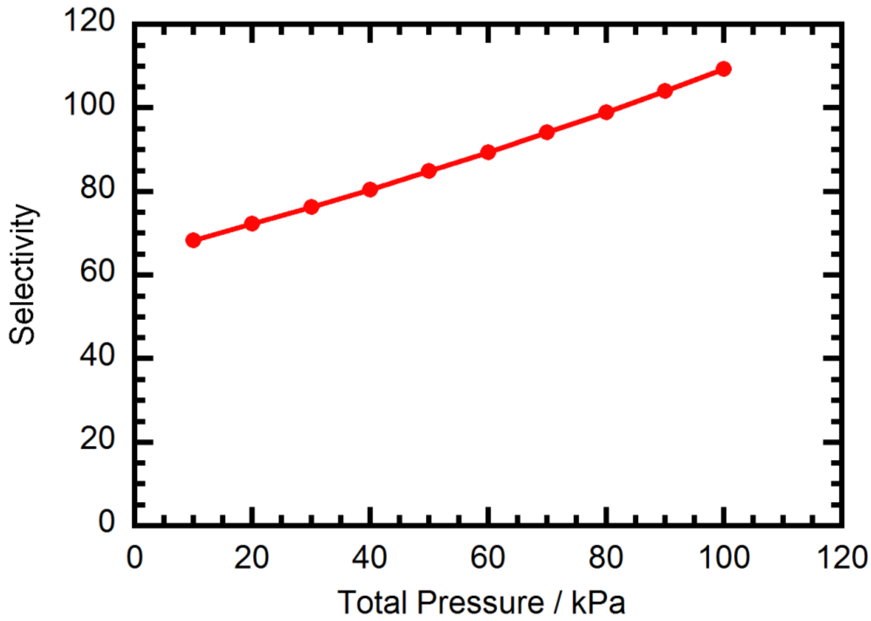

Supplementary Figure 19. Ideal adsorbed solution theory-predicted selectivity for CO_2/Ar in 2 based on the data measured at 298 K for a bulk gas composition of CO_2:Ar = 4:96 (typical composition in the atmosphere).

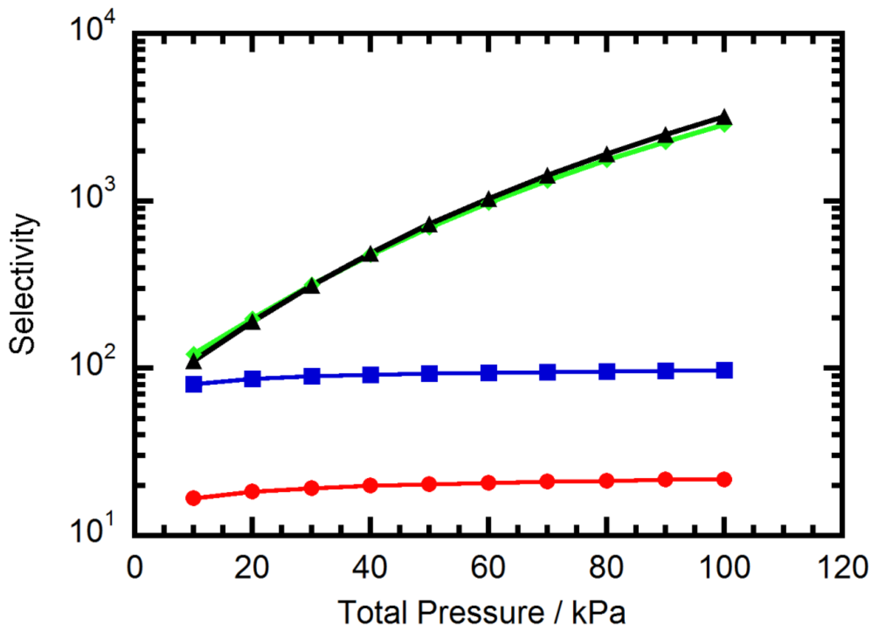

Supplementary Figure 20. Ideal adsorbed solution theory-predicted selectivity for CO_2/CH_4 (red), CO_2/N_2 (blue), CO_2/O_2 (green), and CO_2/Ar (black) in 2 based on the data measured at 298 K for a bulk gas composition of CO_2:X = 50:50.

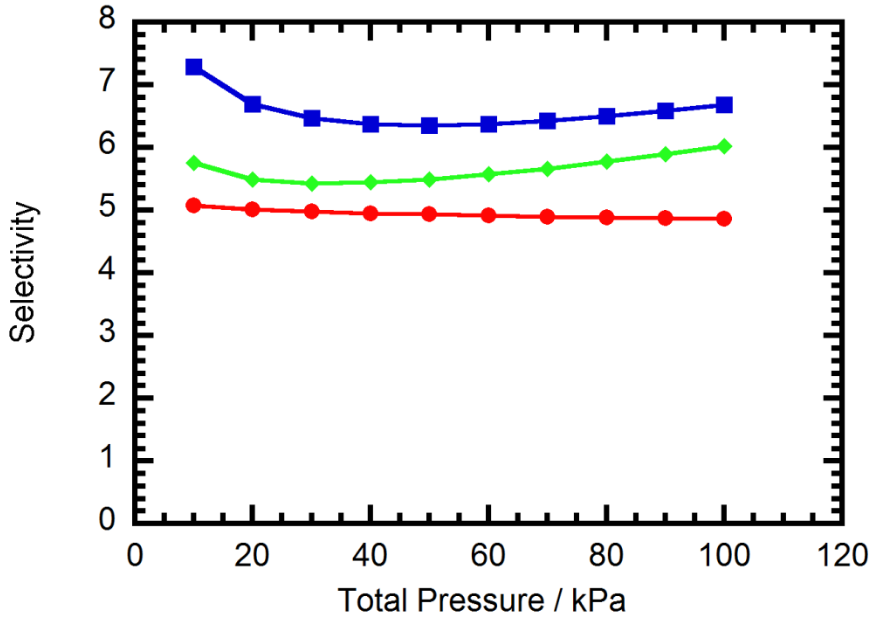

Supplementary Figure 21. Ideal adsorbed solution theory-predicted selectivity for CH_4/N_2 (red), CH_4/O_2 (blue), and CH_4/Ar (green) in 2 based on the data measured at 298 K for a bulk gas composition of CH_4:X = 50:50.

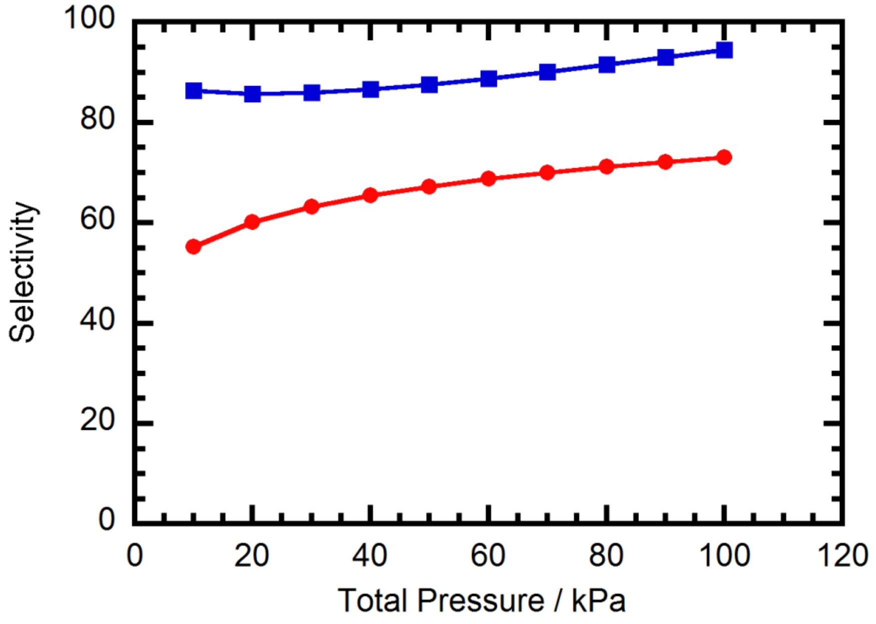

Supplementary Figure 22. Ideal adsorbed solution theory-predicted selectivity for CO_2/N_2 (red) and CO_2/O_2 (blue) in 2 based on the data measured at 298 K for a bulk gas composition of $CO_2:N_2:O_2$ = 1:78:21 (typical composition in the atmosphere).

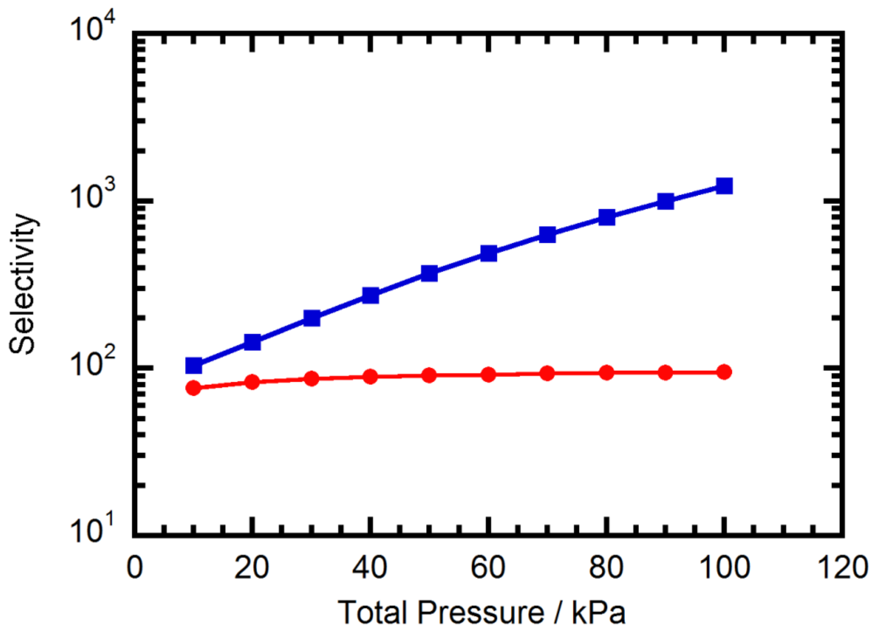

Supplementary Figure 23. Ideal adsorbed solution theory-predicted selectivity for CO_2/N_2 (red) and CO_2/O_2 (blue) in 2 based on the data measured at 298 K for a bulk gas composition of $CO_2:N_2:O_2$ = 1:1:1.

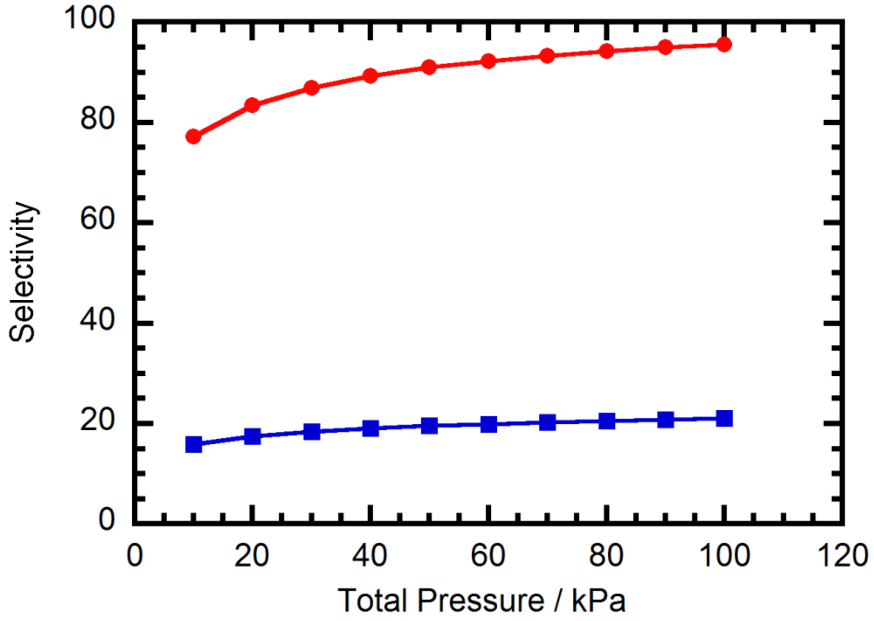

Supplementary Figure 24. Ideal adsorbed solution theory-predicted selectivity for CO_2/N_2 (red) and CO_2/CH_4 (blue) in 2 based on the data measured at 298 K for a bulk gas composition of $CO_2:N_2:CH_4$ = 1:1:1.

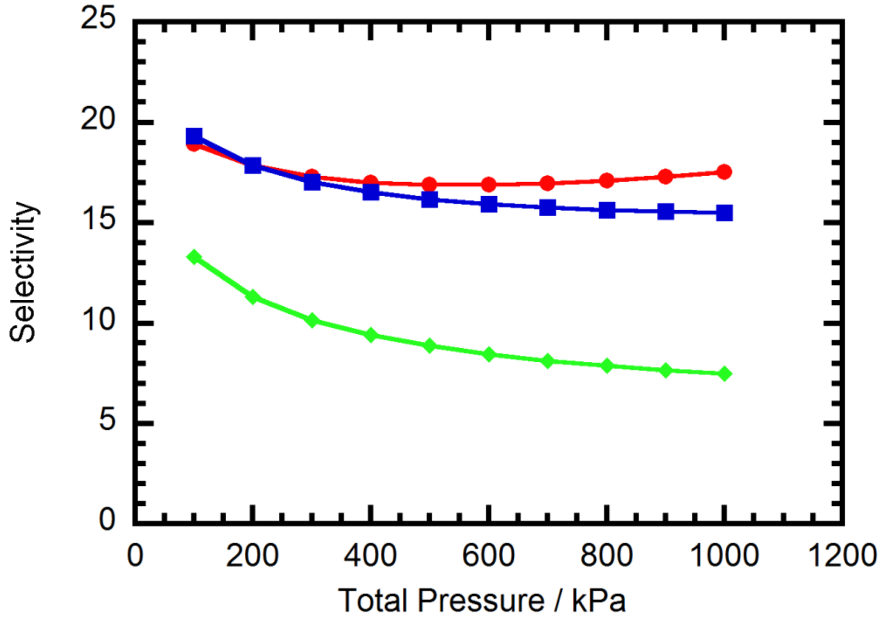

Supplementary Figure 25. Ideal adsorbed solution theory-predicted selectivity for CO_2/CH_4 (red), C_2H_4/CH_4 (blue), and C_2H_6/CH_4 (green) in 2 based on the data measured at 298 K for a bulk gas composition of X:CH_4 = 50:50.

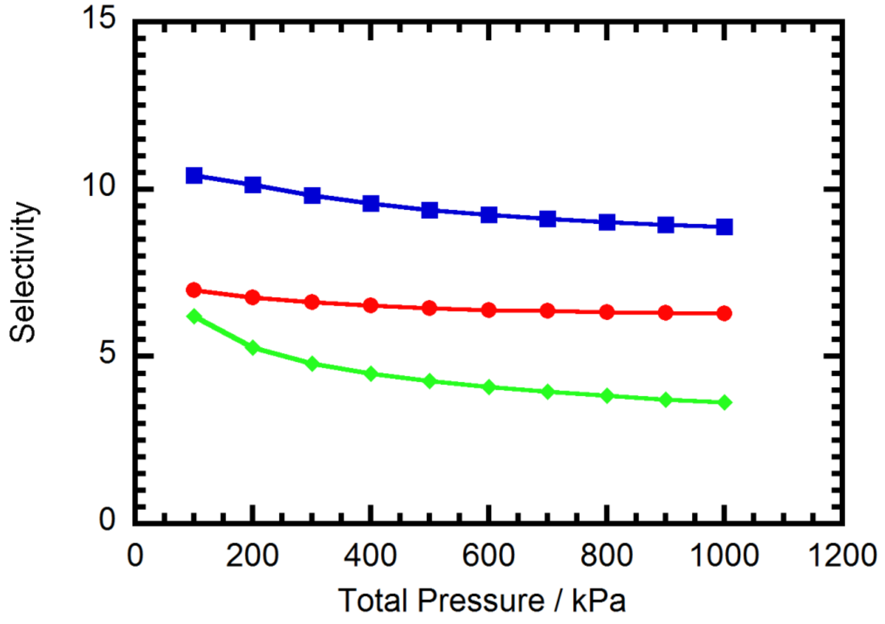

Supplementary Figure 26. Ideal adsorbed solution theory-predicted selectivity for CO_2/CH_4 (red), C_2H_4/CH_4 (blue), and C_2H_6/CH_4 (green) in 3 based on the data measured at 298 K for a bulk gas composition of X:CH_4 = 50:50.

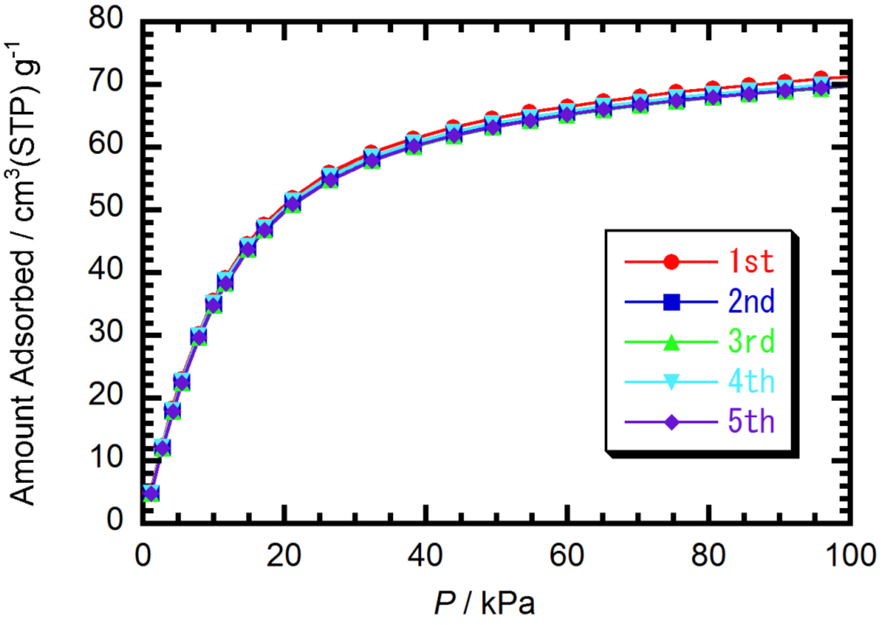

Supplementary Figure 27. Repeated CO_2 adsorption isotherms for 2 at 298 K.

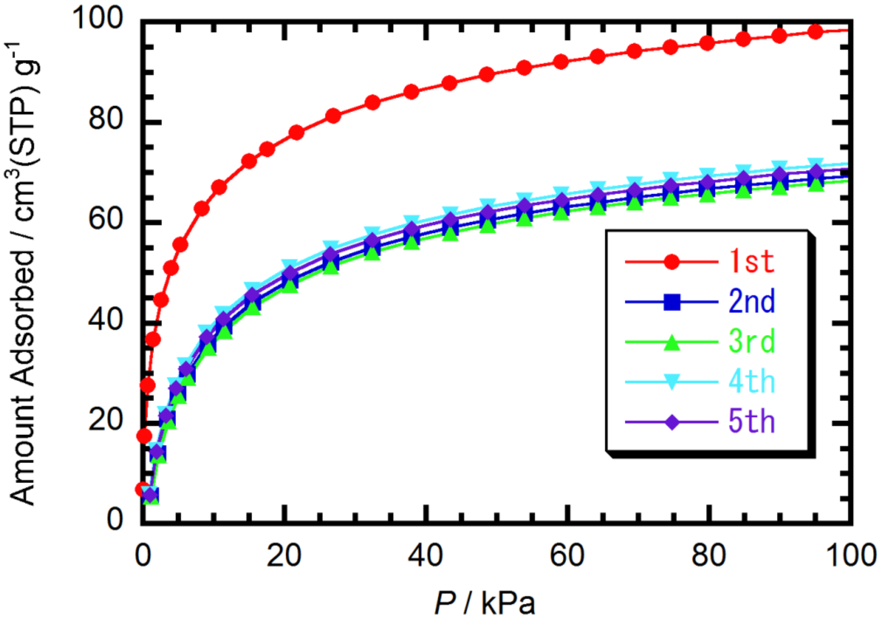

Supplementary Figure 28. Repeated CO_2 adsorption isotherms for Molecular Sieves 13X at 298 K.

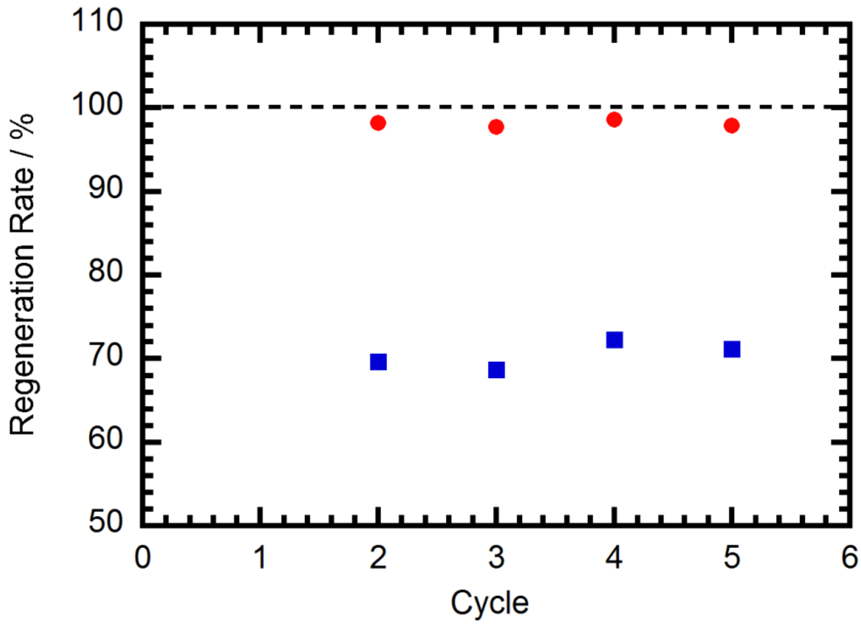

Supplementary Figure 29. Regeneration rate as a function of CO_2 adsorption cycle for 2 (red circles) and Molecular Sieves 13X (blue squares) at 298 K. Rate was calculated according to the equation, (amount adsorbed at 100 kPa in the *n*th cycle)/(amount adsorbed at 100 kPa in the first cycle) × 100 (1).

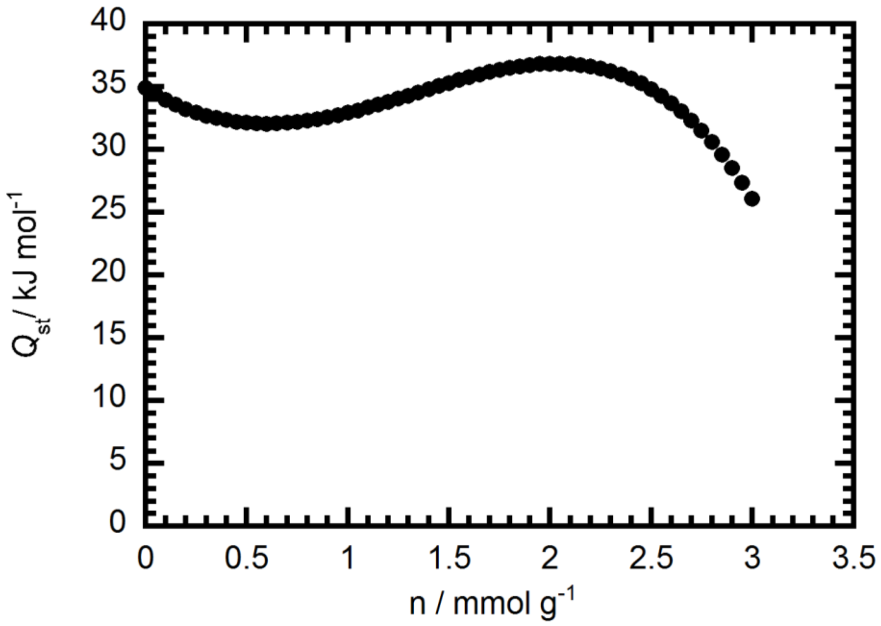

Supplementary Figure 30. Isosteric heat of CO_2 adsorption for 2 at different CO_2 loadings.

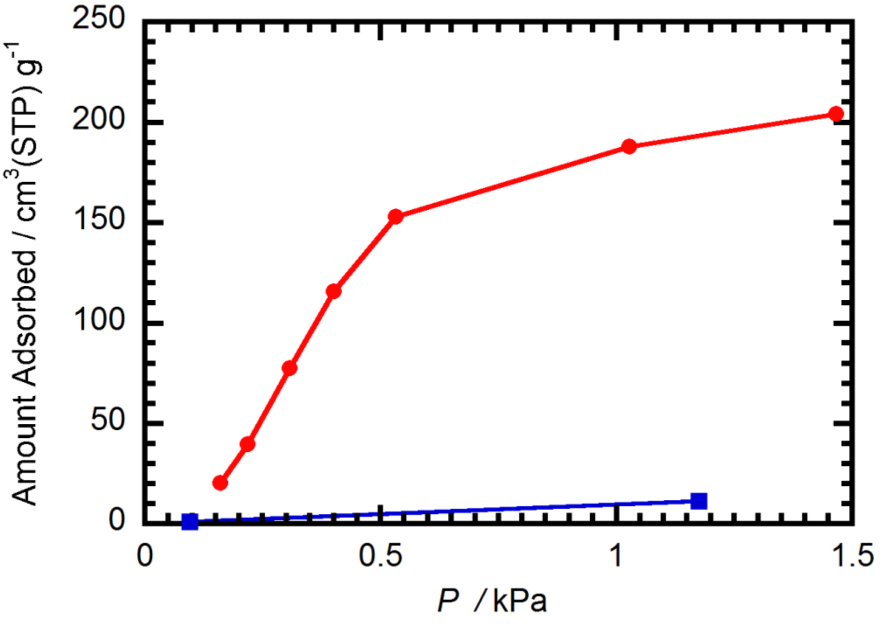

Supplementary Figure 31. H_2O adsorption isotherms for 2 (red) and 3 (blue) at 298 K.

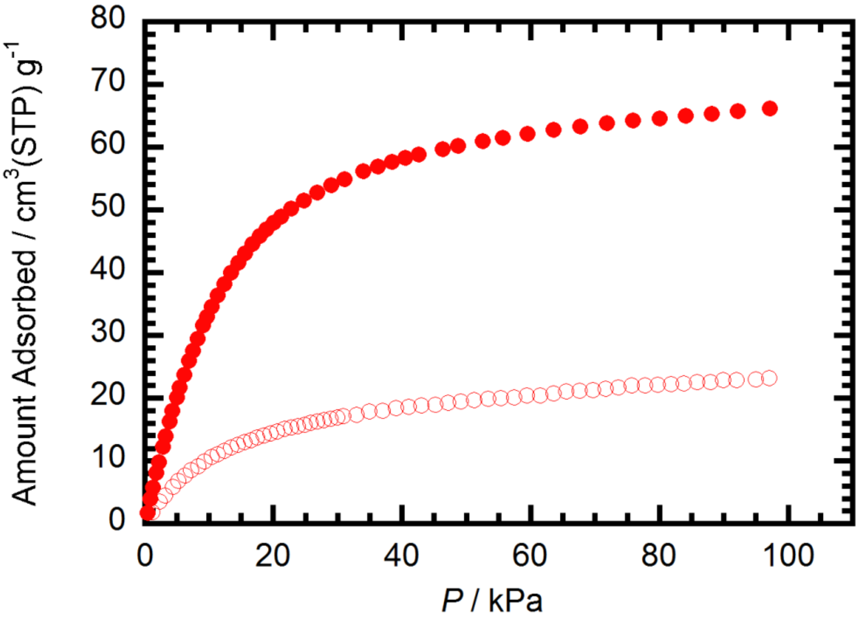

Supplementary Figure 32. CO_2 adsorption isotherms for 2 at 298 K before (closed symbols) and after (open symbols) the H_2O adsorption experiment. The sample used in the H_2O adsorption experiment was activated at 373 K under vacuum before the CO_2 adsorption experiment.

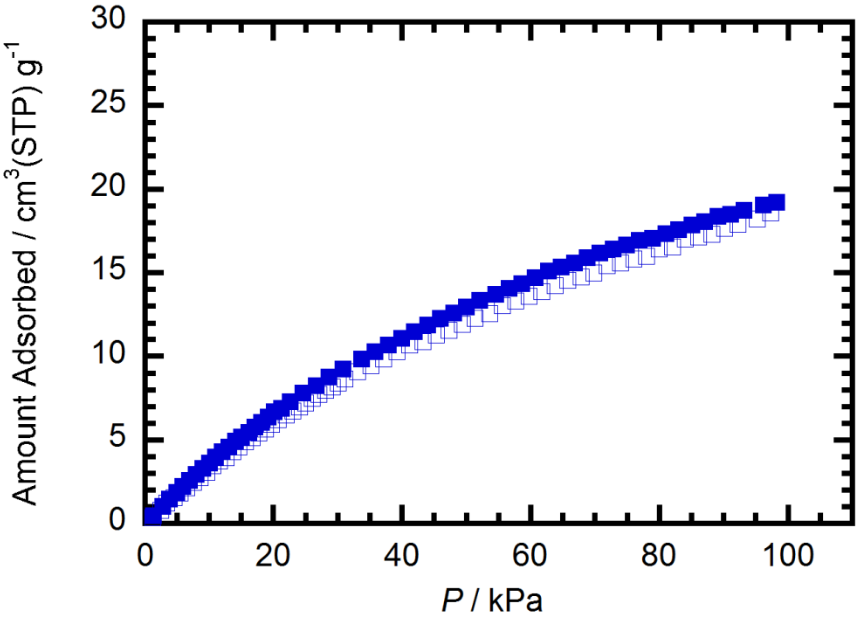

Supplementary Figure 33. CO_2 adsorption isotherms for 3 at 298 K before (closed symbols) and after (open symbols) the H_2O adsorption experiment. The sample used in the H_2O adsorption experiment was activated at 373 K under vacuum before the CO_2 adsorption experiment.

a

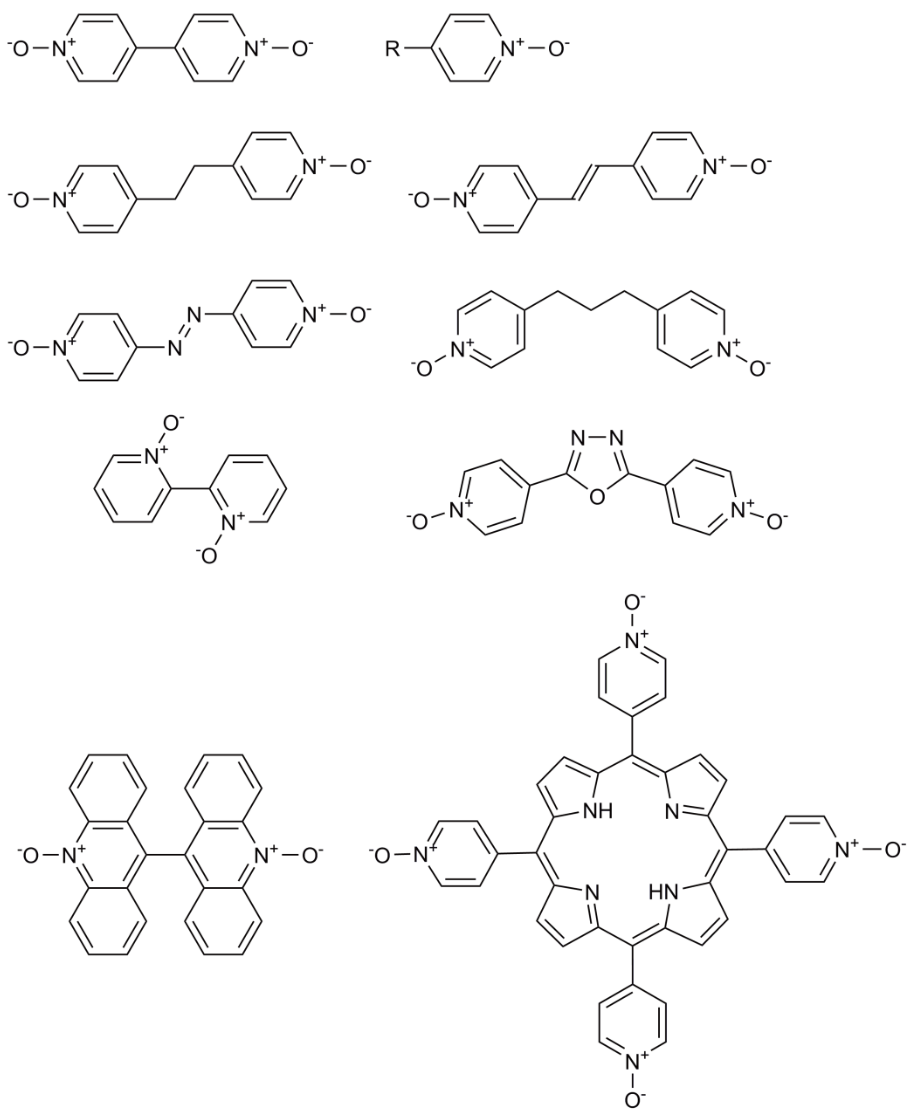

b

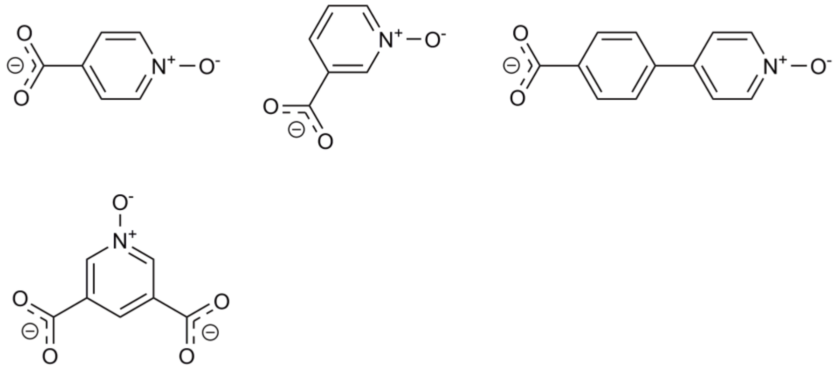

Supplementary Figure 34. List of a variety of (a) neutral and (b) anionic organic ligands with pyridine-*N*-oxide moieties.

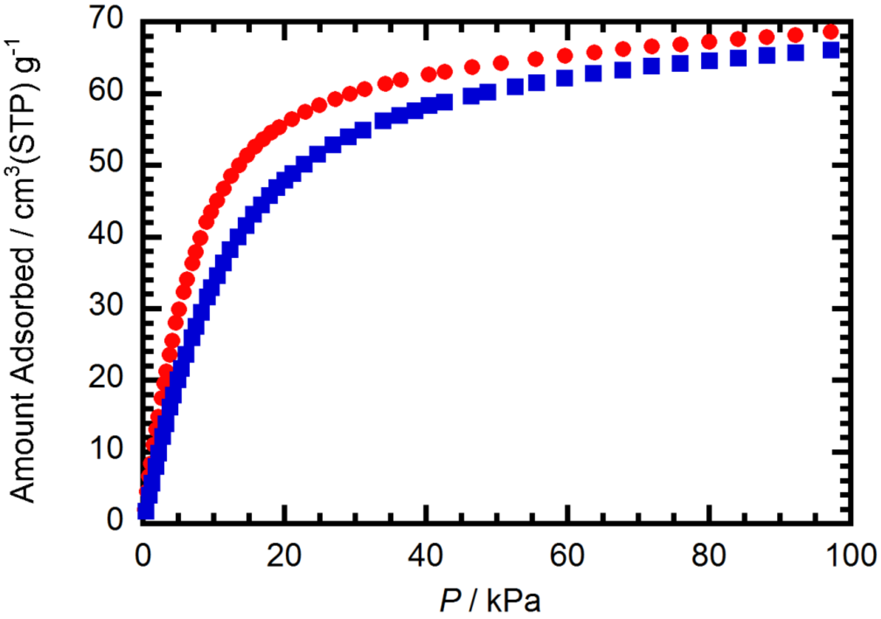

Supplementary Figure 35. **CO_2 adsorption isotherms for 2 at 288 (red) and 298 K (blue).**

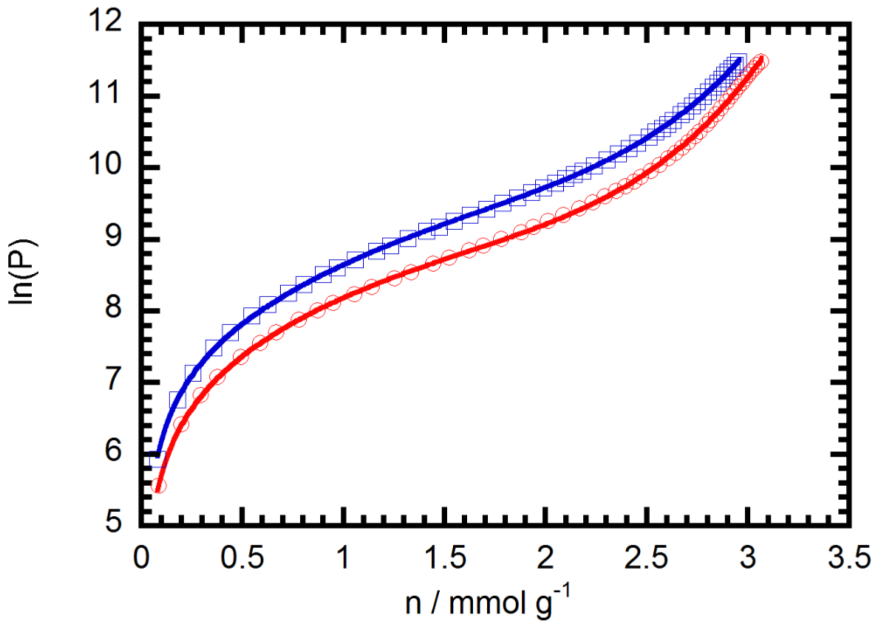

Formula: ln(p) = ln(n) + (1/T) * (a0 + a1 * n + a2 * n^2 + a3 * n^3 + a4 * n^4) + (b0 + b1 * n + b2 * n^2 + b3 * n^3) (2)

Parameters:

	Estimate	Std. Error	t value	Pr(>|t|)
a0	-4197.7542	128.1875	-32.747	< 2e-16 ***
a1	1243.9025	334.2748	3.721	0.000334 ***
a2	-1271.5517	237.4644	-5.355	5.85e-07 ***
a3	237.4548	49.4254	4.804	5.72e-06 ***
a4	29.1541	1.0656	27.359	< 2e-16 ***
b0	22.6080	0.4368	51.754	< 2e-16 ***
b1	-4.4859	1.1364	-3.947	0.000151 ***
b2	5.0529	0.8025	6.297	9.12e-09 ***
b3	-1.2431	0.1632	-7.616	1.83e-11 ***

Signif. codes: 0 ‘***’ 0.001 ‘**’ 0.01 ‘*’ 0.05 ‘.’ 0.1 ‘ ’ 1

Residual standard error: 0.01388 on 96 degrees of freedom

Number of iterations to convergence: 1

Achieved convergence tolerance: 8.935e-06

Supplementary Figure 36. Virial analysis of the CO_2 adsorption isotherms for 2 at 288 (red) and 298 K (blue). The solid lines are the fitting curves using parameters of a_n and b_n.

Supplementary Table 1. Observed and calculated N–O bond distances of 1, 2⊃2DMF, 3⊃0.5DMF, bpdo, and $[Mg_2(bza)_4(OH)_4(bpdo)(H_2O)_8]$ (DMF = *N*,*N*-dimethylformamide, bza^{-} = benzoate, and bpdo = 4,4′-bipyridine-*N*,*N*′-dioxide).

	**1**	**2**⊃2DMF	**3**⊃0.5DMF
N–O bond distance (Å)	1.336(5)	1.338(3)	1.23(2), 1.26(2), 1.28(2), 1.30(2)
	bpdo	$[Mg_2(bza)_4(OH)_4(bpdo)(H_2O)_8]$	
N–O bond distance (Å)	1.284	1.330	

Supplementary Table 2. Top candidates for flue gas separation using five adsorbent evaluation criteria among 2 and other adsorbents.[a,b,c,d]

Adsorbent 298 K	N_1^{ads}	ΔN_1	R	α_{12}^{ads}	S	Reason[e]
ZIF-78	0.60	0.58	**96.3**	34.5	**396**	E
**2**	1.51	1.33	**88.1**	61.8	**314**	E
$[Cu(PF_6)_2(4,4'\text{-}bpy)_2]_n$	0.30	0.27	**90.0**	38.6	**214**	B, F
Zeolite-5A	*3.50*	*2.36*	*67.4*	*61.8*	*163*	*D*
Zeolite-13X	*2.49*	*1.35*	*54.2*	*86.2*	*128*	*D*
ZIF-82	0.41	0.38	**92.5**	26.4	105	E
Co-carborane MOF-4b	0.07	0.06	**83.8**	**154**	104	B
ZIF-81	0.27	0.25	**93.4**	22.7	101	E
Ni-MOF-74	**4.34**	**3.20**	**73.7**	41.1	83.5	C
ZIF-79	0.26	0.24	**92.9**	21.3	83.0	E
ethylenediamine-$H_3[(Cu_4Cl)_3(BTTri)_8]_n$	0.45	0.26	57.6	58.4	77.2	E
$[Zn_2(tcpb)\{p\text{-}(CF_3)NC_5H_4\}_2]_n$	0.16	0.13	**80.7**	43.9	57.9	B, E
Activated carbon Norit R1 Extra	0.38	0.28	**73.7**	10.7	5.09	A

[a] We used the values listed in the Supplementary References 1 and 2 for adsorbents except for **2**. [b] Mixture composition: CO_2/N_2 = 10:90; pressures: p^{ads} = 100 kPa and p^{des} = 10 kPa. [c] N_1^{ads} = CO_2 uptake at adsorption condition (mol kg^{-1}); ΔN_1 (= $N_1^{ads} - N_1^{des}$) = working CO_2 capacity (mol·kg^{-1}); R (= $(\Delta N_1/N_1^{ads}) \times 100$) = regenerability (%); α_{12}^{ads} (= $(N_1^{ads}/N_2^{ads})(y_2/y_1)$) = selectivity at adsorption condition; S (= $((\alpha_{12}^{ads})^2/(\alpha_{12}^{des}))(\Delta N_1/\Delta N_2)$) = sorbent selection parameter. [d] Bold numbers indicate that some materials surpass zeolites. [e] A: simple physical interaction; B: pore size effect; C: open metal sites: D: alkali-metal cations and framework reduction; E: polar groups; F: inorganic anions.

Supplementary Table 3. **Low-pressure adsorption and desorption data for 2 at 298 K.**

CO_2		CH_4		N_2	
Pressure (kPa)	Amount Adsorbed (cm^3(STP) g^{-1})	Pressure (kPa)	Amount Adsorbed (cm^3(STP) g^{-1})	Pressure (kPa)	Amount Adsorbed (cm^3(STP) g^{-1})
0.37800	1.7923	1.5188	0.41049	1.3222	0.058150
0.86570	3.9971	3.2689	0.91570	2.6456	0.11520
1.2550	5.7282	5.2713	1.4564	3.6555	0.16090
1.7852	8.0505	7.2860	2.0213	4.6776	0.25650
2.2044	9.8677	9.3169	2.5692	5.6834	0.35620
2.7864	12.237	11.356	3.1296	6.7217	0.37250
3.2504	13.995	13.383	3.6538	7.7275	0.40770
3.8487	16.273	15.426	4.1921	8.7374	0.46600
4.3168	17.975	17.445	4.7108	9.7432	0.56540
4.9680	20.127	19.488	5.2195	10.769	0.63230
5.4483	21.683	21.539	5.7122	11.779	0.68500
6.0913	23.701	25.581	6.7123	12.801	0.72610
6.8768	26.001	29.659	7.6966	13.803	0.80480
7.4304	27.596	33.757	8.6033	14.829	0.84660
8.1833	29.516	37.807	9.5310	15.851	0.87400
9.0665	31.623	41.914	10.386	16.849	0.94530
9.6648	32.973	45.951	11.276	17.875	0.98720
10.450	34.600	50.058	12.046	18.889	1.0338
11.342	36.433	54.103	12.901	19.911	1.0887
12.327	38.244	61.735	14.287	20.909	1.1464
13.389	40.011	69.793	15.781	22.839	1.2782
14.508	41.632	77.994	17.163	24.883	1.3540
15.599	43.162	86.110	18.505	26.899	1.4866
16.722	44.550	94.335	19.696	28.935	1.5817
17.846	45.825	103.33	21.087	30.971	1.6905
18.949	46.972			33.980	1.8875
20.072	47.989			36.057	1.9691
21.171	48.930			38.056	2.1132

Supplementary Table 3 (continued).

CO_2		CH_4		N_2	
Pressure (kPa)	Amount Adsorbed (cm^3(STP) g^{-1})	Pressure (kPa)	Amount Adsorbed (cm^3(STP) g^{-1})	Pressure (kPa)	Amount Adsorbed (cm^3(STP) g^{-1})
22.726	50.184			40.092	2.2030
24.683	51.551			42.132	2.3686
26.767	52.849			44.160	2.4971
28.932	53.980			46.184	2.6045
31.057	54.966			48.216	2.6875
33.918	56.188			50.260	2.7854
36.267	56.968			52.276	2.8869
38.387	57.666			55.847	3.1459
40.451	58.336			59.891	3.3814
42.591	58.856			63.951	3.5859
46.348	59.738			68.006	3.7901
48.684	60.267			72.046	4.0328
52.559	61.015			76.122	4.2206
55.579	61.560			80.182	4.4651
59.502	62.203			84.246	4.6264
63.585	62.822			88.297	4.8793
67.740	63.342			92.361	5.0318
71.782	63.838			97.407	5.3406
75.905	64.283				
80.019	64.615				
84.061	64.987				
88.151	65.390				
92.209	65.746				
97.207	66.149				
93.113	65.833	89.667	19.019	90.900	4.9429
88.123	65.430	81.910	17.900	84.034	4.6144
84.061	65.087	73.896	16.541	78.052	4.3372

Supplementary Table 3 (continued).

CO_2		CH_4		N_2	
Pressure (kPa)	Amount Adsorbed (cm^3(STP) g^{-1})	Pressure (kPa)	Amount Adsorbed (cm^3(STP) g^{-1})	Pressure (kPa)	Amount Adsorbed (cm^3(STP) g^{-1})
80.036	64.666	65.874	15.166	72.144	4.0184
75.982	64.263	57.811	13.708	66.268	3.6373
71.997	63.833	49.842	12.149	60.331	3.2996
67.956	63.351	45.214	11.258	54.414	3.0162
63.947	62.810	41.144	10.409	48.513	2.6672
59.938	62.275	37.127	9.5610	44.038	2.4352
55.913	61.692	33.069	8.6947	40.088	2.2175
51.916	60.982	28.963	7.7906	36.130	2.0183
47.919	60.269	25.019	6.8813	32.197	1.7893
44.687	59.540	20.998	5.9245	28.267	1.5544
42.490	59.036	16.928	4.9478	25.246	1.3968
38.818	58.091	14.966	4.4482	22.293	1.2783
36.527	57.438	12.935	3.9509	19.333	1.1244
34.541	56.702	10.929	3.4007	18.197	1.0665
32.547	55.994	8.9140	2.8883	16.254	0.93280
30.638	55.109	5.0841	1.8562	15.245	0.83900
28.631	54.230	3.7207	1.4990	14.255	0.76910
26.710	53.216	2.1537	1.0295	13.261	0.74650
24.761	52.091	1.2488	0.76541	12.268	0.67600
22.815	50.856	0.72196	0.61404	11.282	0.59320
20.939	49.466			10.297	0.53090
19.140	47.826			9.3116	0.50280
17.748	46.404			4.5717	0.22730
16.572	45.146			2.2506	0.082570
15.574	43.899				
14.594	42.601				
13.637	41.245				
12.685	39.785				

Supplementary Table 3 (continued).

CO_2		CH_4		N_2	
Pressure (kPa)	Amount Adsorbed (cm^3(STP) g^{-1})	Pressure (kPa)	Amount Adsorbed (cm^3(STP) g^{-1})	Pressure (kPa)	Amount Adsorbed (cm^3(STP) g^{-1})
11.777	38.222				
10.882	36.518				
9.9985	34.673				
8.1670	30.484				
6.8524	26.829				
5.8349	23.886				
5.1633	21.743				
4.6872	20.197				
4.0522	17.951				
3.5231	15.987				
3.0958	14.240				
2.7335	12.701				
2.3917	11.364				
2.1027	10.171				
1.8870	9.0733				
1.6631	8.1474				
1.4759	7.3125				
1.3009	6.5796				
1.1640	5.9148				
1.0367	5.3262				
0.92590	4.8014				

Supplementary Table 3 (continued).

O_2		Ar	
Pressure (kPa)	Amount Adsorbed (cm^3(STP) g^{-1})	Pressure (kPa)	Amount Adsorbed (cm^3(STP) g^{-1})
1.3140	0.047318	1.3059	0.073610
2.6456	0.053674	2.6456	0.13770
3.6799	0.091771	3.6677	0.12710
4.6531	0.16050	4.6613	0.20720
5.6915	0.17540	5.6834	0.23740
6.7217	0.21950	6.7095	0.26820
7.7235	0.26560	7.7316	0.29830
8.7415	0.30080	8.7333	0.37940
9.7554	0.35410	9.7391	0.46120
10.782	0.41620	10.769	0.49400
11.791	0.44460	11.775	0.55340
12.801	0.47820	12.789	0.62260
13.807	0.53160	13.803	0.69170
14.833	0.56750	14.813	0.76680
15.859	0.60400	15.839	0.81050
16.873	0.63810	16.869	0.83420
17.871	0.69680	17.855	0.92540
18.889	0.75200	18.873	0.99470
19.903	0.80650	19.891	1.0475
20.937	0.81650	20.921	1.0736
22.851	0.92660	22.843	1.1992
24.899	1.0181	24.887	1.2835
26.915	1.1381	26.911	1.3715
28.972	1.2015	28.943	1.4660
30.963	1.3376	30.946	1.6107
34.001	1.5055	33.984	1.7942
36.049	1.5756	36.049	1.8839
38.077	1.6820	38.069	2.0262

Supplementary Table 3 (continued).

O_2		Ar	
Pressure (kPa)	Amount Adsorbed (cm^3(STP) g^{-1})	Pressure (kPa)	Amount Adsorbed (cm^3(STP) g^{-1})
40.109	1.7685	40.096	2.1419
42.141	1.8763	42.108	2.2654
44.177	1.9496	44.168	2.3419
46.180	2.0616	46.180	2.4784
48.228	2.1443	48.220	2.5986
50.260	2.2172	50.260	2.6400
52.288	2.3372	52.272	2.7791
55.851	2.5012	55.851	2.9615
59.923	2.6743	59.903	3.1524
63.979	2.9125	63.942	3.3397
68.043	3.1329	68.019	3.5247
72.095	3.3485	72.066	3.7309
76.142	3.5763	76.122	3.9028
80.227	3.6956	80.170	4.0992
84.262	3.8252	84.242	4.2638
88.338	3.9993	88.297	4.4364
92.365	4.1696	92.382	4.6632
97.460	4.3762	97.411	4.9618
90.830	4.1234	90.859	4.6904
83.944	3.8378	83.957	4.4237
78.011	3.6205	78.068	4.1882
72.082	3.4252	72.103	4.0118
66.178	3.1544	66.211	3.7497
60.253	2.9471	60.282	3.5114
54.361	2.6549	54.402	3.2028
48.448	2.4330	48.477	2.9383
43.989	2.2404	44.022	2.7513

Supplementary Table 3 (continued).

O_2		Ar	
Pressure (kPa)	Amount Adsorbed (cm^3(STP) g^{-1})	Pressure (kPa)	Amount Adsorbed (cm^3(STP) g^{-1})
40.052	2.0878	40.064	2.5304
36.106	1.9051	36.106	2.4082
32.148	1.7539	32.184	2.2167
28.206	1.5570	28.239	2.0129
25.217	1.4170	25.225	1.8688
22.249	1.3211	22.273	1.7274
19.292	1.1791	19.313	1.5838
18.176	1.1142	18.193	1.5022
16.246	1.0147	16.259	1.3893
15.224	0.96500	15.224	1.3238
14.239	0.94300	14.235	1.3000
13.237	0.91770	13.233	1.2609
12.276	0.84380	12.276	1.1877
11.270	0.81080	11.291	1.1233
10.293	0.74670	10.285	1.0846
9.2953	0.73560	9.2953	1.0535
4.5147	0.53420	4.5635	0.76050
2.2140	0.38220	2.2588	0.59070
1.1056	0.26480	1.1458	0.44720
0.55230	0.22380	0.58620	0.38620

Supplementary Table 4. High-pressure adsorption and desorption data for 2 at 298 K (pore volume = 0.31 cm^3 g^{-1}).

CO_2			CH_4		
Pressure (kPa)	Excess Uptake (mmol g^{-1})	Total Uptake (mmol g^{-1})	Pressure (kPa)	Excess Uptake (mmol g^{-1})	Total Uptake (mmol g^{-1})
0.17086	0.016054	0.016075	0.50039	0.0022330	0.0022957
13.972	0.84868	0.85043	32.589	0.16606	0.17014
39.704	1.2450	1.2500	65.312	0.30637	0.31455
71.574	1.4179	1.4269	96.740	0.40946	0.42158
102.98	1.5201	1.5330	128.64	0.50283	0.51896
135.09	1.6015	1.6185	160.82	0.58500	0.60518
167.69	1.6699	1.6910	193.21	0.65559	0.67984
200.39	1.7326	1.7579	226.11	0.71993	0.74833
233.19	1.7921	1.8216	259.22	0.77669	0.80927
266.16	1.8466	1.8804	292.39	0.82777	0.86454
299.14	1.9012	1.9392	325.68	0.87346	0.91444
332.16	1.9550	1.9973	392.39	0.94961	0.99904
398.11	2.0541	2.1049	459.35	1.0150	1.0729
464.19	2.1497	2.2091	526.63	1.0720	1.1385
530.49	2.2389	2.3071	594.13	1.1211	1.1962
597.12	2.3159	2.3929	661.79	1.1650	1.2488
663.96	2.3887	2.4747	729.60	1.2048	1.2973
730.86	2.4581	2.5531	797.37	1.2434	1.3446
797.97	2.5209	2.6250	865.44	1.2735	1.3834
865.22	2.5791	2.6924	923.06	1.2976	1.4150
922.45	2.6230	2.7441			
853.98	2.5716	2.6834	852.22	1.2739	1.3821
785.44	2.5220	2.6244	781.81	1.2412	1.3404
717.13	2.4576	2.5507	712.56	1.2078	1.2981
649.46	2.3902	2.4743	643.03	1.1712	1.2525
582.52	2.3203	2.3954	573.75	1.1299	1.2024
515.77	2.2377	2.3040	505.50	1.0828	1.1466

Supplementary Table 4 (continued).

CO_2			CH_4		
Pressure (kPa)	Excess Uptake (mmol g^{-1})	Total Uptake (mmol g^{-1})	Pressure (kPa)	Excess Uptake (mmol g^{-1})	Total Uptake (mmol g^{-1})
449.54	2.1504	2.2080	438.01	1.0273	1.0826
383.70	2.0540	2.1029	370.92	0.96270	1.0094
311.52	1.9398	1.9794	293.58	0.87223	0.90915
241.63	1.8242	1.8548	221.65	0.76553	0.79337
173.88	1.7005	1.7224	154.03	0.63371	0.65303
109.11	1.5550	1.5687	90.122	0.46346	0.47476
43.185	1.2900	1.2955	34.505	0.23738	0.24170
22.677	1.0722	1.0750	12.673	0.12253	0.12412
12.752	0.83513	0.83672	4.8452	0.073403	0.074009
8.4272	0.65596	0.65702	1.9380	0.051513	0.051755
6.0319	0.52069	0.52145	0.84536	0.040242	0.040347
4.5068	0.41698	0.41755			
3.4560	0.33624	0.33667			
2.6991	0.27257	0.27291			
2.1347	0.22192	0.22219			
1.7005	0.18143	0.18164			
1.3660	0.14878	0.14895			
1.1029	0.12235	0.12249			
0.89436	0.10087	0.10098			
C_2H_4			C_2H_6		
Pressure (kPa)	Excess Uptake (mmol g^{-1})	Total Uptake (mmol g^{-1})	Pressure (kPa)	Excess Uptake (mmol g^{-1})	Total Uptake (mmol g^{-1})
0.12880	0.019267	0.019283	0.13187	0.016930	0.016947
14.050	0.85221	0.85397	17.036	0.73567	0.73780
40.732	1.2178	1.2229	46.845	0.98257	0.98845
72.793	1.3866	1.3957	80.452	1.0927	1.1028

Supplementary Table 4 (continued).

C_2H_4			C_2H_6		
Pressure (kPa)	Excess Uptake (mmol g^{-1})	Total Uptake (mmol g^{-1})	Pressure (kPa)	Excess Uptake (mmol g^{-1})	Total Uptake (mmol g^{-1})
104.88	1.4690	1.4822	113.75	1.1375	1.1518
137.66	1.5338	1.5511	147.28	1.1743	1.1929
170.79	1.5878	1.6094	181.05	1.2064	1.2294
204.21	1.6341	1.6599	214.90	1.2353	1.2626
237.80	1.6739	1.7041	248.81	1.2612	1.2929
271.48	1.7094	1.7439	282.90	1.2849	1.3211
305.36	1.7406	1.7795	316.98	1.3077	1.3484
373.05	1.7860	1.8337	385.08	1.3400	1.3896
440.78	1.8271	1.8836	453.30	1.3693	1.4281
508.87	1.8618	1.9274	521.54	1.3959	1.4639
576.97	1.8936	1.9683	589.82	1.4223	1.4996
645.31	1.9195	2.0034	658.23	1.4452	1.5320
713.60	1.9440	2.0371	726.66	1.4692	1.5656
782.04	1.9652	2.0677	795.09	1.4917	1.5978
850.48	1.9860	2.0979	863.53	1.5123	1.6283
918.61	2.0038	2.1253	922.80	1.5287	1.6533
847.38	1.9947	2.1062	852.07	1.5257	1.6400
777.27	1.9814	2.0832	781.37	1.5195	1.6237
707.85	1.9666	2.0589	710.71	1.5109	1.6050
637.76	1.9499	2.0327	640.44	1.5004	1.5847
568.57	1.9303	2.0039	570.73	1.4884	1.5632
499.78	1.9061	1.9705	501.38	1.4746	1.5399
431.41	1.8791	1.9344	432.50	1.4595	1.5154
363.59	1.8472	1.8936	364.28	1.4404	1.4873
289.20	1.8041	1.8409	289.40	1.4161	1.4531
216.86	1.7489	1.7764	216.87	1.3851	1.4127

Supplementary Table 4 (continued).

C_2H_4			C_2H_6		
Pressure (kPa)	Excess Uptake (mmol g^{-1})	Total Uptake (mmol g^{-1})	Pressure (kPa)	Excess Uptake (mmol g^{-1})	Total Uptake (mmol g^{-1})
147.93	1.6754	1.6941	147.51	1.3448	1.3634
82.740	1.5607	1.5711	80.642	1.2783	1.2884
32.956	1.3495	1.3537	30.794	1.1342	1.1381
14.882	1.1384	1.1403	13.090	0.96697	0.96861
8.5749	0.97537	0.97644	7.2044	0.83590	0.83680
5.7254	0.85279	0.85351	4.6553	0.73860	0.73918
4.1650	0.75849	0.75901	3.3087	0.66490	0.66532
3.2028	0.68360	0.68400	2.5042	0.60693	0.60725
2.5594	0.62250	0.62282	1.9782	0.56003	0.56028
2.1008	0.57163	0.57189	1.6103	0.52129	0.52149
1.7613	0.52855	0.52877	1.3402	0.48864	0.48881
1.5000	0.49157	0.49175	1.1361	0.46082	0.46096
1.2958	0.45941	0.45957	0.97487	0.43675	0.43687
1.1300	0.43131	0.43145			
0.99401	0.40647	0.40660			

Supplementary Table 5. High-pressure adsorption and desorption data for 3 at 298 K (pore volume = 0.20 cm^3 g$^{–1}$).

CO_2			CH_4		
Pressure (kPa)	Excess Uptake (mmol g$^{–1}$)	Total Uptake (mmol g$^{–1}$)	Pressure (kPa)	Excess Uptake (mmol g$^{–1}$)	Total Uptake (mmol g$^{–1}$)
0.40849	0.0058853	0.0059183	0.54954	0.00071964	0.00076403
27.073	0.32569	0.32788	34.913	0.070066	0.072886
56.472	0.55649	0.56106	69.421	0.13575	0.14137
85.353	0.71623	0.72315	102.28	0.18889	0.19717
115.28	0.84769	0.85705	135.17	0.24079	0.25173
145.95	0.95603	0.96790	168.23	0.29059	0.30421
177.28	1.0494	1.0639	201.23	0.33659	0.35289
209.01	1.1285	1.1455	234.45	0.37921	0.39821
241.15	1.1966	1.2163	267.68	0.42155	0.44326
273.54	1.2590	1.2813	301.04	0.45980	0.48423
306.14	1.3144	1.3395	367.56	0.52661	0.55647
371.84	1.4036	1.4341	434.32	0.58786	0.62319
437.98	1.4819	1.5181	501.28	0.64302	0.68384
504.68	1.5467	1.5885	568.36	0.69662	0.74295
571.52	1.6064	1.6539	635.63	0.74557	0.79744
638.80	1.6541	1.7074	702.99	0.79072	0.84816
706.16	1.7013	1.7605	770.42	0.83539	0.89842
773.71	1.7429	1.8080	838.06	0.87505	0.94369
841.43	1.7780	1.8490	905.70	0.91160	0.98587
909.09	1.8176	1.8945			
840.24	1.7860	1.8569	834.57	0.88628	0.95463
771.79	1.7509	1.8157	764.27	0.85596	0.91848
703.82	1.7100	1.7690	694.81	0.82061	0.87737
636.07	1.6641	1.7172	625.90	0.78107	0.83215
568.54	1.6128	1.6600	557.60	0.73761	0.78306
501.57	1.5549	1.5964	489.77	0.68910	0.72897
434.95	1.4890	1.5249	422.62	0.63545	0.66982

Supplementary Table 5 (continued).

CO_2			CH_4		
Pressure (kPa)	Excess Uptake (mmol g^{-1})	Total Uptake (mmol g^{-1})	Pressure (kPa)	Excess Uptake (mmol g^{-1})	Total Uptake (mmol g^{-1})
368.94	1.4129	1.4433	355.82	0.57681	0.60571
298.56	1.3144	1.3388	280.18	0.50201	0.52474
230.54	1.1908	1.2097	209.20	0.41884	0.43579
165.63	1.0347	1.0482	141.53	0.32612	0.33757
104.01	0.82320	0.83164	76.432	0.22048	0.22665
44.722	0.49475	0.49837	33.203	0.12179	0.12447
24.925	0.32375	0.32576	10.827	0.067494	0.068368
11.424	0.17374	0.17467	3.6347	0.046397	0.046691
5.5532	0.094438	0.094887	1.2965	0.036829	0.036934
2.7963	0.052640	0.052866	0.52405	0.031377	0.031419
1.4472	0.030269	0.030386			
0.76884	0.018032	0.018094			
C_2H_4			C_2H_6		
Pressure (kPa)	Excess Uptake (mmol g^{-1})	Total Uptake (mmol g^{-1})	Pressure (kPa)	Excess Uptake (mmol g^{-1})	Total Uptake (mmol g^{-1})
0.35550	0.0080228	0.0080514	0.37204	0.0050259	0.0050560
24.230	0.42955	0.43151	28.929	0.26922	0.27156
51.898	0.72427	0.72847	60.522	0.43465	0.43956
81.232	0.88515	0.89174	92.099	0.53536	0.54285
111.58	1.0117	1.0208	123.96	0.61841	0.62851
142.79	1.1122	1.1238	156.27	0.68819	0.70096
174.67	1.1962	1.2104	189.07	0.74621	0.76169
206.92	1.2662	1.2831	222.10	0.79663	0.81486
239.63	1.3275	1.3472	255.35	0.84030	0.86132
272.53	1.3823	1.4046	288.89	0.87771	0.90155
305.63	1.4299	1.4550	322.45	0.91335	0.94004

Supplementary Table 5 (continued).

C_2H_4			C_2H_6		
Pressure (kPa)	Excess Uptake (mmol g^{-1})	Total Uptake (mmol g^{-1})	Pressure (kPa)	Excess Uptake (mmol g^{-1})	Total Uptake (mmol g^{-1})
372.20	1.5016	1.5323	389.85	0.96383	0.99627
439.22	1.5622	1.5986	457.47	1.0070	1.0452
506.46	1.6149	1.6571	525.22	1.0449	1.0891
573.98	1.6601	1.7081	593.20	1.0768	1.1270
641.66	1.6993	1.7531	661.43	1.1044	1.1607
709.56	1.7350	1.7947	729.61	1.1328	1.1952
777.45	1.7691	1.8349	797.87	1.1581	1.2268
845.62	1.7957	1.8675	866.26	1.1788	1.2538
913.71	1.8255	1.9034	923.57	1.1986	1.2790
842.80	1.8181	1.8897	853.83	1.1912	1.2651
772.16	1.8071	1.8724	783.44	1.1801	1.2474
702.29	1.7928	1.8519	713.60	1.1674	1.2284
632.82	1.7745	1.8275	644.05	1.1508	1.2055
563.66	1.7507	1.7977	575.17	1.1311	1.1797
495.36	1.7208	1.7619	506.72	1.1080	1.1506
427.79	1.6844	1.7198	438.50	1.0797	1.1163
360.59	1.6401	1.6698	370.82	1.0455	1.0764
286.45	1.5764	1.5999	296.43	0.99581	1.0203
216.54	1.4927	1.5104	225.46	0.92930	0.94782
150.01	1.3798	1.3920	157.50	0.83614	0.84901
87.173	1.2059	1.2130	93.211	0.69858	0.70616
39.915	0.90823	0.91146	40.202	0.48547	0.48873
18.952	0.64622	0.64775	16.212	0.31710	0.31841
10.423	0.47458	0.47542	7.3388	0.22256	0.22315
6.2150	0.36421	0.36471	3.5867	0.17097	0.17126
3.8876	0.29250	0.29281	1.8735	0.14182	0.14197

Supplementary Table 5 (continued).

C_2H_4			C_2H_6		
Pressure (kPa)	Excess Uptake (mmol g^{-1})	Total Uptake (mmol g^{-1})	Pressure (kPa)	Excess Uptake (mmol g^{-1})	Total Uptake (mmol g^{-1})
2.5209	0.24467	0.24487	1.0444	0.12446	0.12455
1.6859	0.21196	0.21210	0.62285	0.11342	0.11347
1.1657	0.18889	0.18899			
0.83401	0.17197	0.17204			

Supplementary Table 6. Fit parameters used in this study, where *n* is the gas uptake in mmol g^{-1}.

2					
(1) CO_2 (0~100 kPa, single-site Langmuir-Freundlich model)					
a	b	c			
3.1631	0.06185	1.1701			
(2) CH_4 (0~100 kPa, single-site Langmuir-Freundlich model)					
a	b	c			
2.9785	0.004174	1.0144			
(3) N_2 (0~100 kPa, single-site Langmuir-Freundlich model)					
a	b	c			
3.1259	0.00073226	1.0322			
(4) O_2 (0~100 kPa, single-site Langmuir-Freundlich model)					
a	b	c			
0.83101	0.0011587	1.2208			
(5) Ar (0~100 kPa, single-site Langmuir-Freundlich model)					
a	b	c			
0.80541	0.0019187	1.1491			
(6) CO_2 (0~1000 kPa, dual-site Langmuir-Freundlich model)					
a	b	c	d	e	f
1.5678	0.073193	1.0476	2.4049	9.7464e-5	1.3507
(7) C_2H_4 (0~1000 kPa, dual-site Langmuir-Freundlich model)					
a	b	c	d	e	f
1.1472	0.075205	1.1573	2.5254	0.014261	0.55657
(8) C_2H_6 (0~1000 kPa, dual-site Langmuir-Freundlich model)					
a	b	c	d	e	f
1.1534	0.099589	0.99107	3.0153	0.00056076	0.86404
(9) CH_4 (0~1000 kPa, single-site Langmuir-Freundlich model)					
a	b	c			
2.1516	0.0038879	0.90615			

Supplementary Table 6 (continued).

**3**					
(1) CO_2 (0~1000 kPa, dual-site Langmuir-Freundlich model)					
a	b	c	d	e	f
3.2573	0.00032816	0.89031	1.7691	0.010276	0.9173
(2) C_2H_4 (0~1000 kPa, dual-site Langmuir-Freundlich model)					
a	b	c	d	e	f
2.1292	0.01002	0.7741	0.48721	0.0052275	1.5575
(3) C_2H_6 (0~1000 kPa, single-site Langmuir-Freundlich model)					
a	b	c			
1.832	0.015721	0.72652			
(4) CH_4 (0~1000 kPa, single-site Langmuir-Freundlich model)					
a	b	c			
2.3049	0.0012045	0.94363			

$$n = \frac{a \times b \times p^c}{1 + b \times p^c} \quad \text{(single-site Langmuir-Freundlich model)} \quad (3)$$

$$n = \frac{a \times b \times p^c}{1 + b \times p^c} + \frac{d \times e \times p^f}{1 + e \times p^f} \quad \text{(dual-site Langmuir-Freundlich model)} \quad (4)$$

Supplementary Table 7. **Crystallographic data of 1, 2⊃2DMF and 3⊃0.5DMF (DMF = *N*,*N*-dimethylformamide).**

	**1**	**2**⊃2DMF	**3**⊃0.5DMF
Formula	$C_{18}H_{20}Mg_2N_2O_{10}$	$C_{32}H_{30}Mg_2N_4O_{12}$	$C_{39}H_{31}Ca_2N_5O_{13}$
Formula weight	472.97	711.22	870.38
Crystal system	Monoclinic	Monoclinic	Monoclinic
Space group	*C*2/*c*	*C*2/*c*	*C*2/*c*
a / Å	22.2003(19)	18.2716(9)	21.47(3)
b / Å	7.3575(7)	17.2585(9)	19.39(3)
c / Å	15.5257(12)	13.5539(7)	10.099(14)
β / °	130.5877(18)	129.6663(13)	119.867(16)
V / Å^3	1925.8(3)	3290.1(3)	3646(9)
Z	4	4	4
Crystal size / mm	0.08 × 0.08 × 0.08	0.60 × 0.20 × 0.20	0.40 × 0.12 × 0.04
T / K	173	173	173
D_c / g cm^{-3}	1.631	1.436	1.563
F_{000}	984.00	1480.00	1776.00
λ / Å	0.71075	0.71075	0.71075
μ(Mo-Kα) / cm^{-1}	1.90	1.44	3.95
Data measured	7193	15818	17682
Data unique	1698	3724	4185
R_{int}	0.0723	0.0224	0.1111
No. of observations	1698	3724	4185
No. of variables	145	228	343
R_1 (I > 2.00σ(I))^a	0.0620	0.0556	0.0984
R (all reflections)^a	0.1188	0.0606	0.1505
wR_2 (all reflections)^b	0.2270	0.1635	0.3305
GOF	1.139	1.049	1.103
CCDC number	997086	997087	997088

^{a} $R_1 = R = \Sigma ||F_o| - |F_c|| / \Sigma |F_o|$. ^{b} $wR_2 = [\Sigma w(F_o^2 - F_c^2)^2 / \Sigma w(F_o^2)^2]^{1/2}$.

Supplementary References

(1) Bae, Y.-S. & Snurr, R. Q. Development and Evaluation of Porous Materials for Carbon Dioxide Separation and Capture. *Angew. Chem., Int. Ed.* **50**, 11586-11596 (2011).

(2) Noro, S. *et al.* Highly Selective CO_2 Adsorption Accompanied with Low-Energy Regeneration in a Two-Dimensional Cu(II) Porous Coordination Polymer with Inorganic Fluorinated PF_6^- Anions. *Inorg. Chem.* **52**, 280-285 (2013).
